# Tuning the Optical Properties of Silicon Quantum Dots via Surface Functionalization with Conjugated Aromatic Fluorophores

**DOI:** 10.1038/s41598-018-21181-8

**Published:** 2018-02-14

**Authors:** Mohammed Abdelhameed, Diego Rota Martir, Shalimar Chen, William Z. Xu, Olabode O. Oyeneye, Subrata Chakrabarti, Eli Zysman-Colman, Paul A. Charpentier

**Affiliations:** 1 Department of Chemical and Biochemical Engineering, Western University, London, Ontario N6A 5B9 Canada; 2 Organic Semiconductor Centre, EaStCHEM School of Chemistry, University of St Andrews, St Andrews, Fife, KY16 9ST UK; 3 Department of Pathology and Laboratory Medicine, Schulich School of Medicine and Dentistry, Western University, London, N6A 5B9 Ontario Canada

## Abstract

Silicon Quantum Dots (SQDs) have recently attracted great interest due to their excellent optical properties, low cytotoxicity, and ease of surface modification. The size of SQDs and type of ligand on their surface has a great influence on their optical properties which is still poorly understood. Here we report the synthesis and spectroscopic studies of three families of unreported SQDs functionalized by covalently linking to the aromatic fluorophores, 9-vinylphenanthrene, 1-vinylpyrene, and 3-vinylperylene. The results showed that the prepared functionalized SQDs had a highly-controlled diameter by HR-TEM, ranging from 1.7–2.1 nm. The photophysical measurements of the assemblies provided clear evidence for efficient energy transfer from the fluorophore to the SQD core. Fӧrster energy transfer is the likely mechanism in these assemblies. As a result of the photogenerated energy transfer process, the emission color of the SQD core could be efficiently tuned and its emission quantum efficiency enhanced. To demonstrate the potential application of the synthesized SQDs for bioimaging of cancer cells, the water-soluble perylene- and pyrene-capped SQDs were examined for fluorescent imaging of HeLa cells. The SQDs were shown to be of low cytotoxicity

## Introduction

Silicon Quantum Dots (SQDs) in the quantum-size range (2–10 nm) have recently attracted great interest due to their unique optoelectronic properties which include broad absorption spectra, high stability against photobleaching, and size-tunable photoluminescence (PL), ranging from visible to near-infrared, by changing their size^1^. These nanoparticles also have excellent biocompatibility^2^, low cytotoxicity^3^, and versatile surface modification capability, and are therefore promising candidates for various biological and biomedical applications, such as bioimaging^4^ and photodynamic therapy^5^.

A number of methods have been reported for the synthesis of SQDs, such as bulk silicon etching^6^, laser pyrolysis^7^, and preparation in supercritical fluids^8^, which produce SQDs with wavelength-tunable emission by controlling their size. A simple solution-based reduction method^9–13^ has been shown advantageous over previous syntheses methods due to its simplicity and ease with which the surface chemistry can be modified^14,15^. Interestingly, the use of aromatic fluorophores for the modification of the SQDs surface is an efficient pathway to both tune the optical properties and improve the colloidal stability of SQDs^16^.

Despite some reports of aromatic fluorophores incorporated into SQDs that permit sensitization of the SQDs via energy transfer, there are still limited examples of luminescent SQDs covalently functionalized with conjugated emissive compounds obtained through a solution-based reduction method^17,18^. Bard *et al*.^19^ used the electrochemical charge injection to induce the luminescence from SQDs passivated with a combination of hydrogen and alkoxide ligands; Rosso-Vasic *et al*.^20^ reported up to 55% efficiency of energy transfer from SQDs to a Ru-based dye. Sommer *et al*.^21^ reported very fast energy transfer in a system composed of SQDs functionalized with vinyl pyridine; Erogbogbo *et al*.^16^ observed improvement in the emission efficiency of SQDs functionalized by anthracene in the hydrophobic core of micelles as a result of energy transfer. Recently, Ceroni *et al*. investigated SQDs covalently linked with pyrene units through a nonconjugated bridge and observed efficient energy transfer from the donor pyrene moieties to the acceptor SQD core^22^. Using a conjugated bridge may offer even improved optical properties through photogenerated energy transfer.

Here, we report the synthesis and surface functionalization of SQDs using a conjugated bridging approach with 9-vinyl phenanthrene, 1-vinyl pyrene, and 3-vinyl perylene to produce SQD-phenanthrene, SQD-pyrene, and SQD- perylene, respectively. Phenanthrene, pyrene, and perylene fluorophores were chosen for the surface passivation of SQDs due to their high stability and excellent optical properties including high fluorescence quantum yield^23^. Functionalization of the SQDS with these fluorophores is expected to improve the quantum yield of SQDs and tunability of PL emission, which can be applied in many fields such as bioimaging. The functionalized SQDs were characterized by UV−Vis absorption spectroscopy, Fourier-transform infrared (FTIR) spectroscopy, steady-state and times-resolved emission spectroscopy, high-resolution transmission electronic microscopy (HRTEM), and X-ray photoelectron spectroscopy (XPS).

## Results and Discussion

**Figure 1:**
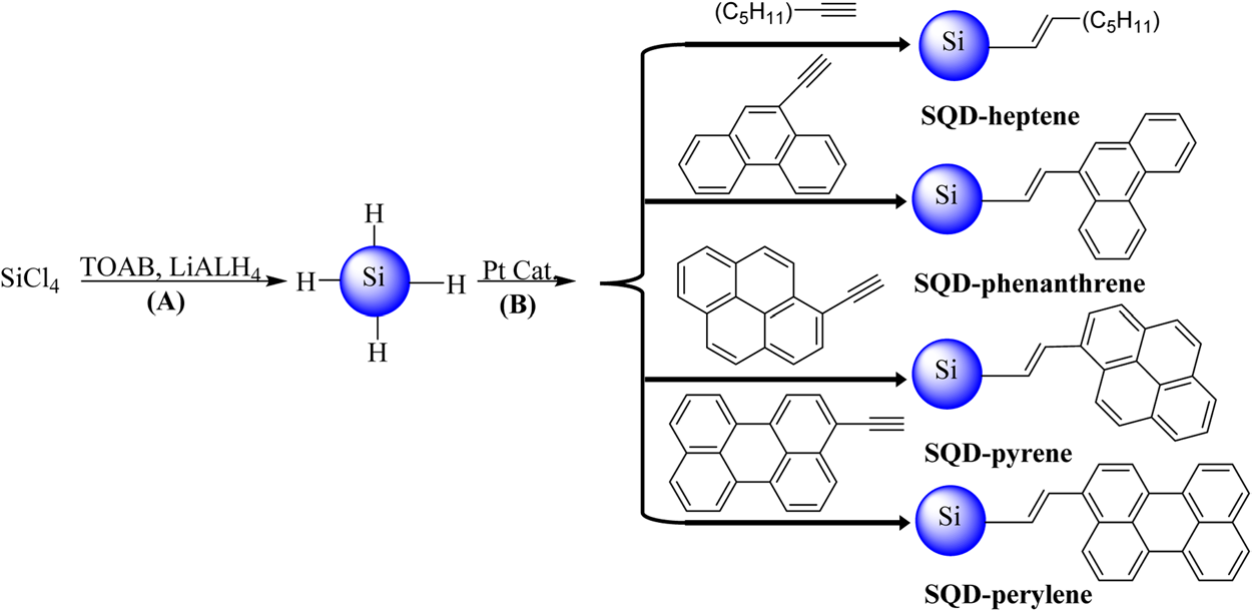
Synthesis route of H-terminated SQDs (**A**) and their surface passivation using different ligands (**B**).

SQDs functionalized with aromatic fluorophores were prepared following a solution-based reduction route as shown in Fig. 1 ^24^. Silicon tetrachloride (SiCl_4_) was used as the silicon source and was reduced by a strong reducing agent (LAH). The resultant H-terminated SQDs were passivated with organic molecules in the presence of a Pt catalyst.

### Size and structure

**Figure 2:**
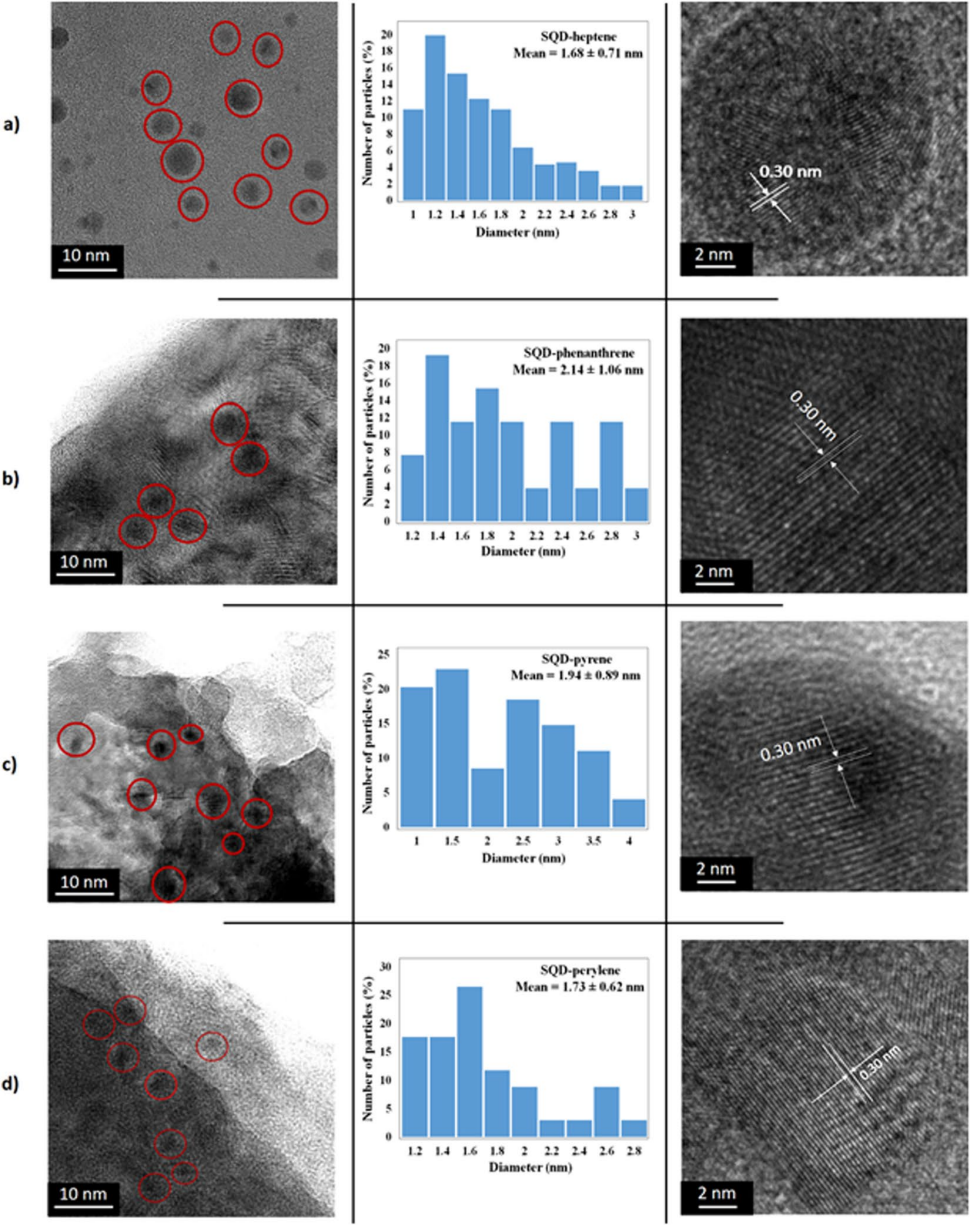
TEM (left), diameter distributions (middle) and HR-TEM images (right) of SQD-heptene (**A**), SQD-phenanthrene (**B**), SQD-pyrene (**C**), and SQD-perylene (**D**).

Figure 2 shows the TEM, size distribution histograms, and HR-TEM of the synthesized SQD-heptene, SQD-phenanthrene, SQD-pyrene, and SQD-perylene assemblies. The TEM images demonstrate that the functionalized SQDs are quasi-spherical without obvious agglomeration and aggregation. The size distributions indicate that the average diameter for SQD-heptene, SQD-phenanthrene, SQD-pyrene, and SQD-perylene are 1.68 ± 0.71, 2.14 ± 1.06, 1.94 ± 0.89, and 1.73 ± 0.62 nm, respectively, after the analysis of more than 150 dots from different regions of the grids. The HR-TEM images display the high crystallinity of the SQDs, as evidenced by the distinct lattice fringes with 0.30 nm interplanar spacing, which is consistent with the (111) plane of diamond silicon^14,25^. It should be mentioned that the low contrast of the TEM and HR-TEM images is due to the extreme small dimensions of SQDs and also the low atomic weight of silicon compared to metallic or semiconductor quantum dots, which results in poor visualization^12,26^.

**Figure 3:**
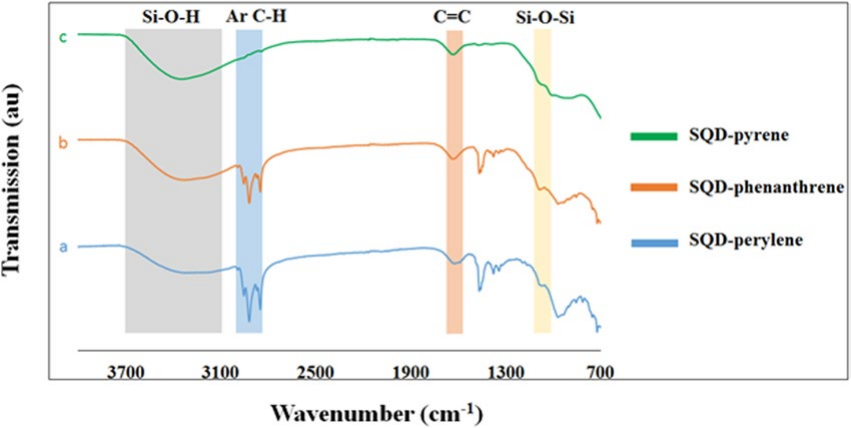
FTIR spectra of SQD-perylene (**a**), SQD-phenanthrene (**b**), and SQD-pyrene (**c**).

To confirm aromatic ligand binding to the surface of SQDs, both FTIR and XPS spectroscopy were performed. Figure 3 shows the FTIR spectra of SQD-phenanthrene, SQD-pyrene, and SQD-perylene. The broad peak at 3010–3680 cm^−1^ can be attributed to OH stretching^27^, and the peak at 1000–1100 cm^−1^ indicates the presence of Si-O-Si stretching. This provides evidence for the oxidation of the surface of the SQDs^28^. The peaks at 3010–2850 and 1416–1480 cm^−1^ are attributed to aromatic fluorophores C-H stretching and bending, respectively^29^. The wide peak at 1630 cm^−1^ is assigned to the stretching vibrations of (-C = C), which was also observed in analogous systems^30,31^. Furthermore, the characteristic peaks of the (≡C-H) and (C≡C) stretching vibrations at 3230–3330 and 2115–2040 cm^−1^, respectively observed for the free alkynyl fluorophores (see Supplementary Fig. S1), as well as the stretching vibration peak of (Si–H) at 2100–2250 cm^−1^ ^32^, are absent in the spectra of the functionalized SQDs. This indicates complete reaction of the alkynyl fluorophores with the H-terminated SQDs.

**Figure 4:**
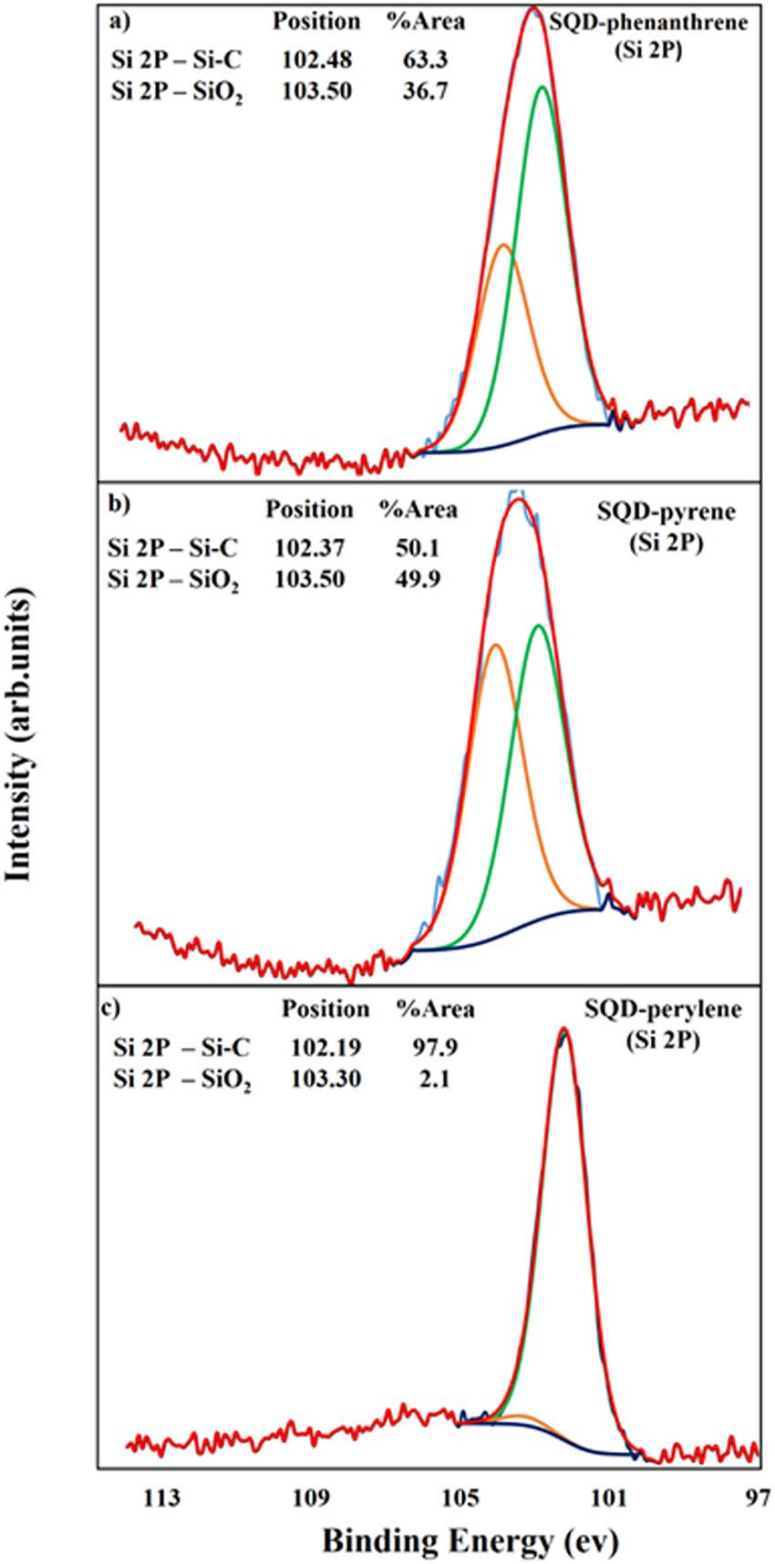
XPS spectra of Si 2p for SQD-phenanthrene (**a**), SQD-pyrene (**b**), and SQD-perylene (**c**).

The dye-terminated SQDs were further investigated using high resolution XPS spectroscopy. Figure 4 shows high resolution XPS spectra of Si 2p for SQD-phenanthrene (a), SQD-pyrene (b), and SQD-perylene (c). The Si 2p spectra in Fig. 4 were fitted with two peaks and one Shirley background. The deconvoluted peaks centred at 102.48, 102.37, and 102.19 eV are attributed to Si-C^33,34^, which confirms that the SQDs surface changed from H- to organic dye termination. The components at 103.50 and 103.30 eV are assigned to Si-O, indicating that the surface of the SQDs has been partially oxidized under ambient conditions^34^. XPS spectrum of C 1 s for SQD-pyrene (see Supplementary Fig. S2) shows multiple peaks binding energy assigned to C-Si, C = C, CO_3_, O-C = O, C = O, along with a key feature located at 291.4 eV, which is attributed to the characteristic shake-up peak, exhibited by the conjugated system or aromatic groups^35^. This further confirms the bonding of pyrene to SQDs through a conjugated linkage. These XPS results are in agreement with the FTIR results, confirming that the passivation of the SQDs has been successfully achieved and the aromatic fluorophores are covalently bonded to SQDs through a conjugated linkage.

### Photophysical properties

The photophysical properties, including emission lifetime (τ_e_), quantum efficiency, (Φ_PL_) and emission and excitation spectra of the SQD-phenanthrene, SQD-perylene, SQD-pyrene, reference SQD-heptene, and chromophores phenanthrene, perylene and pyrene were investigated in DCM and are summarized in Table 1.

**Table 1:** Photophysical properties collected in DCM at 298 K.

	λ_ex_ nm	λ_em_ nm(RI)^*a*^	Φ_PL_^b^ %^*a,c*^	τ_e_ ns(pe)^*f*^
**perylene**	360	465 (1), 489 (0.95), 530 (0.41)	89	4.4
**pyrene**	360	384 (1), 404 (0.39), 431 (0.13)	61	2.2 (9), 17.9 (91)
**phenanthrene**	315	358 (0.74), 367 (0.94), 376 (1), 387 (0.69), 392 (0.48)	14	1.4 (67), 6.3 (33)
**SQD-heptene**	360	446	8	1.8 (27), 6.07 (73)
**SQD-heptene**	315	374	—	—
**SQD-perylene**	360	515	18^c^	2.5 (38), 6.6 (62)
**SQD-pyrene**	360	396 (1), 415 (0.95), 482 (0.52)	11^*d*^	1.9 (46), 7.7 (54)
**SQD-phenanthrene**	315	439	8^*e*^	1.8 (58), 6.9 (42)

^a^RI = relative intensity of the emission peak. ^b^Quinine sulfate employed as the external reference (Φ_PL_ = 54.6% in 0.5 M H_2_SO_4_ at 298 K, λ_exc_ = 360 nm)^44^. ^c^λ_ex_ = 440 nm, ^d^λ_ex_ = 330 nm, ^e^λ_ex_ = 310 nm. ^*f*^ pe = pre-exponential weighting factor, in relative % intensity, of the emission decay kinetics (λ_ex_ = 378 nm).

**Figure 5:**
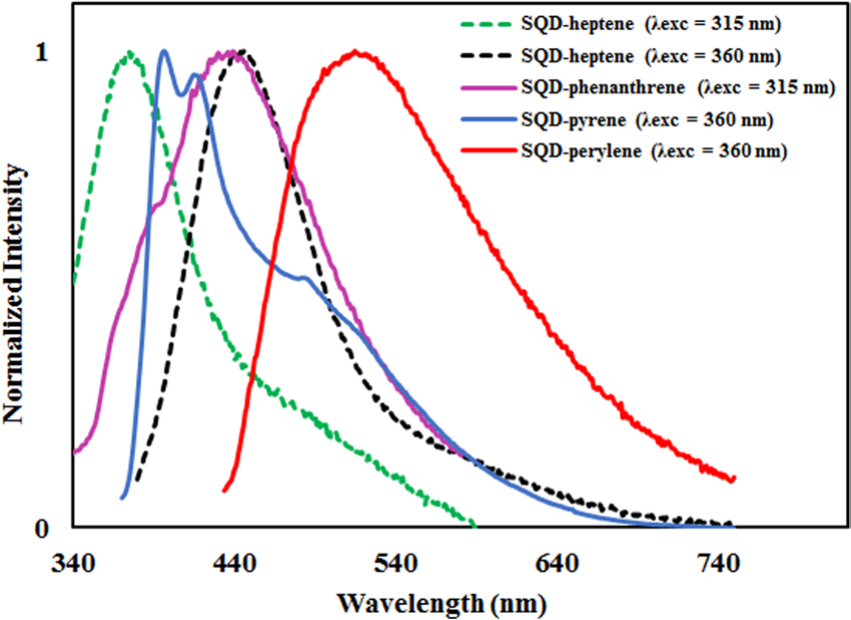
Emission spectra of SQD-heptene (dotted green line, λ_ex_ = 315 nm), SQD-phenanthrene (solid pink line, λ_ex_ = 315 nm), SQD-heptene (dotted black line, λ_ex_ = 360 nm), SQD-pyrene (solid blue line, λ_ex_ = 360 nm), and SQD-perylene (solid red line, λ_ex_ = 360 nm) collected in degassed DCM at 298 K.

SQD-perylene and SQD-pyrene exhibited broad, red- and blueshifted emission maxima at λ_max_ = 515 and 396 nm, respectively with modestly higher photoluminescence quantum yields, Φ_PL_, (SQD-perylene: Φ_PL_ = 18%, SQD-pyrene: Φ_PL_ = 11%) compared to the heptene-capped control counterpart SQDs (SQD-heptene: λ_max_ = 446 nm, Φ_PL_ = 8%), when excited at 360 nm. Similarly, the SQD-phenanthrene exhibited a red-shifted emission maximum at λ_max_ = 439 nm compared to SQD-heptene (λ_max_ = 374 nm) when excited at 315 nm, but with a Φ_PL_ of 8%, which is similar to that of SQD-heptene (Fig. 5).

In water, both the SQD-pyrene and SQD-perylene exhibited an emission peak maxima at 395 and 521 nm, respectively (see Supplementary Fig. S21). Evidence of pyrene excimer emission at concentrations higher than 10 and 176 μM was observed for SQD-pyrene and free pyrene at 487 nm, respectively (see Supplementary Figures S11 and S12)^36^. It is also worth noting that SQD-phenanthrene, SQD-pyrene, and SQD-perylene exhibited red-shifted emissions comparing to their corresponding free aromatic fluorophores (see Supplementary Table 1 and Figures S5–S7). Hence, depending on the surface functionalization of the SQD core, different emission maxima were observed, pointing to an ability of the chromophores to tune the emission properties of the nanoparticle.

Generally, any σ-π conjugation between Si atoms and π-conjugated fluorophores should increase the interaction of the aromatic fluorophores with the electronic wave functions of the SQDs^34^, influencing the electronic structure of SQDs and inducing red-shifted emissions compared to fluorophore-free SQDs. This may account for the observed significant red-shifted emission of SQD-perylene and SQD-phenanthrene by 69 and 65 nm, respectively, compared to that of SQD-heptene. However, the emission of SQD-pyrene did not behave in an analogous manner. The deviation from the expected behavior may be attributed to the high ratio of Si-O_x_ species, (SiO_2:_ Si-C ~ 1:1), on the silicon network, which seems crucial for influencing the optical properties. This is in good agreement with a previous report of blue shifting in the emission of SQDs that have a monolayer of organic molecules grafted to their surface via controlled thermal or photoinitiated surface oxidation^37^. From the XPS spectra, the ratio of SiO_2_ on the silicon surface in SQD-pyrene and SQD-perylene was found to be (25:1), respectively. Thus, the blue shift in the emission of SQD-pyrene compared to that of SQD-heptene when excited at wavelength of 360 nm may have resulted from a greater surface oxidation.

The photoluminescence spectra for the SQD-perylene, SQD-pyrene, and SQD-phenanthrene assemblies were collected by photoexciting either at 360 nm, where the SQD core predominantly absorbs, or at 450 nm, 340 nm and 315 nm, where the perylene, pyrene or phenanthrene moieties, respectively, are predominantly photoexcited. Identical photoluminescence spectra without any contribution from the free perylene, pyrene or phenanthrene were observed (see Supplementary Figures S8–S10). Thus, SQD-perylene, SQD-pyrene, and SQD-phenanthrene assemblies exhibit efficient energy transfer from the fluorophore to the SQD core upon photoexcitation. As no triplet states are involved in the energy transfer process between the fluorophore and the nanoparticle core as evident from the recorded lifetimes given in Table 1, and the PL spectra of all donor fluorophores exhibit spectral overlap with SQDs absorption (see Supplementary Figures S5–S7), Fӧrster energy transfer is the likely mechanism for energy transfer in these systems^38,39^. Photoluminescence excitation spectra measured by detecting the emissions of SQD-fluorophore are further consistent with energy transfer from the fluorophore to the SQD core, with the appearance of the corresponding perylene, pyrene and phenanthrene-based absorptions (see Supplementary Figure S5–S7). Energy transfer from pyrene to SQDs in SQD-pyrene is also evidenced in the emission lifetime decay monitored at 420 nm collected upon photoexcitation at 378 nm. Indeed, the bi-exponential emission lifetime of SQD-pyrene of 1.9 and 7.7 ns is similar to that of SQD-heptene (τ_e_ = 1.8, 6.07 ns) and much shorter than that of the free pyrene fluorophore (τ_e_ = 2.2 and 17.9 ns) (see Supplementary Figures S10 and S11). The shorter lifetime of SQD-pyrene compared to free pyrene is consistent with energy transfer from the bound pyrene to the SQD core. Energy transfer from pyrene moieties covalently linked to silicon nanocrystals was also previously observed^22^. Similarly, SQD-perylene and SQD-phenanthrene exhibited respectively bi-exponential emission decays of τ_e_ = 2.5, 6.6 ns and τ_e_ = 1.8, 6.9 ns, which are correspondingly slightly shorter and longer compared to those of the free chromophores perylene (τ_e_ = 4.4 ns) and phenanthrene (τ_e_ = 1.4, 6.3 ns).

It is worth noting that because of the small size of the functionalized SQDs, the PL deviates significantly from the quantum confinement prediction. This is attributed to the non-radiative trapping of electrons in the higher excited states by surface states^24^. Thus, the nature of the fluorophore linked to the SQD surface, and the electronic communication between SQD core and fluorophores (through the σ-π conjugation) strongly influence the optical properties of the SQD core. This strategy is beneficial for producing SQDs with tunable emission wavelengths by modifying the surface with aromatic fluorophores and can be applied to overcome current synthetic limitations where only blue-emitting nanoparticles are predominantly obtained.

### Fluorescent cellular imaging study

**Figure 6:**
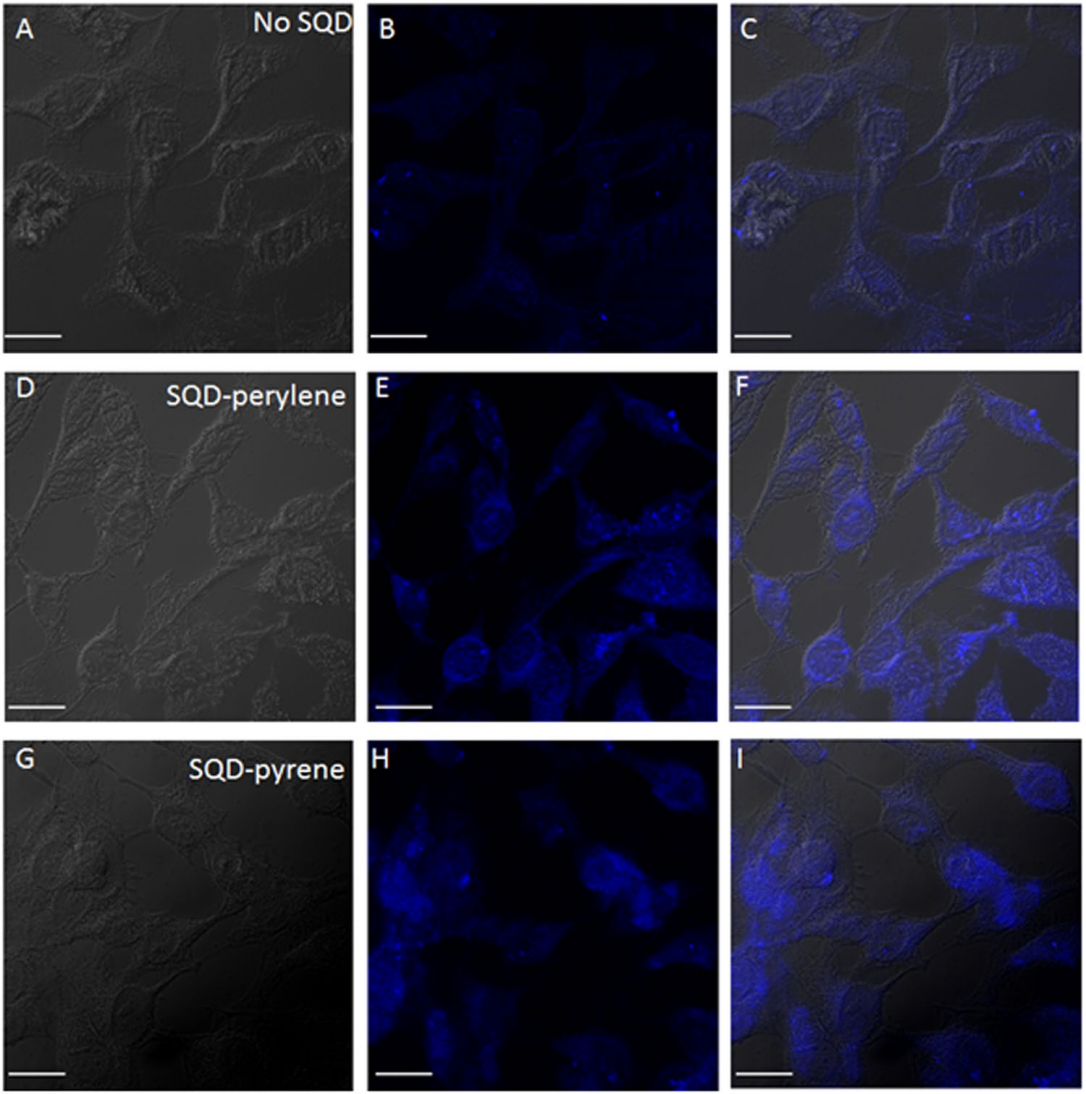
Confocal images of HeLa cells for DIC images (panels A, D, and G), fluorescence images (panels B, E, and H), and merged images (panels C, F, and I). Scale bar = 10 μm. Panels (A–C) are for control sample without SQDs, (**D**–**F**) and (**G**–**I**) are for cells incubated with SQD-perylene and SQD-pyrene, respectively.

To demonstrate the suitability of SQD-perylene and SQD-pyrene assemblies for bioimaging application, they were used for *in vitro* fluorescent imaging of cancerous HeLa cells. The cells were incubated for 3 hours with SQD-perylene and SQD-pyrene, respectively. The nanoparticles were then excited at 405 nm using confocal microscopy to monitor the uptake of SQDs (Fig. 6). The images were collected after 135 seconds of constant excitation to quench the cellular autofluorescence. Fluorescence imaging of HeLa cells without SQDs (panels A–C), with SQD-perylene (panels D–F), and SQD-pyrene (panels G–I) show bright fluorescence for cells incubated with SQD. The efficient uptake of nanoparticles by cells demonstrates the potential utility of SQDs for bioimaging studies.

### Cytotoxicity Studies

**Figure 7:**
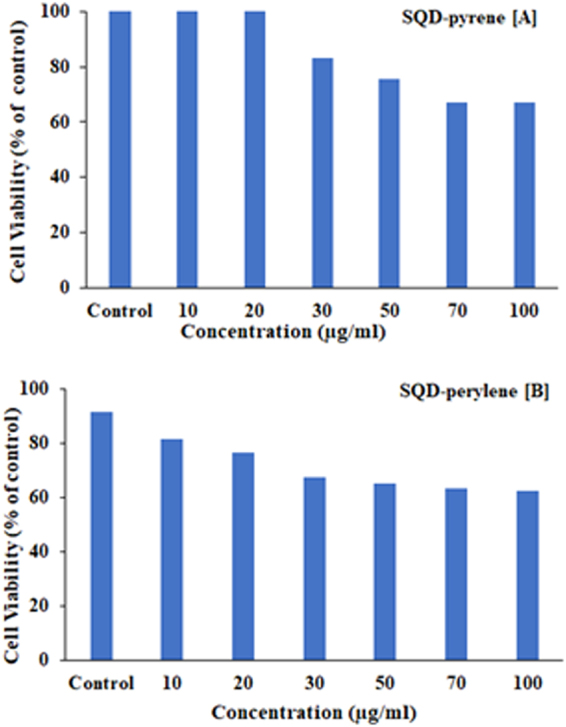
Cytotoxicity effect of the nanoparticles SQD-pyrene (**A**) and SQD-perylene (**B**) measured by TNF-α assay.

The cytotoxicity of SQD-pyrene and SQD-perylene was assessed by TNF-α cytotoxicity assay to investigate whether the materials would be harmful when used for biomedical applications. Figure 7 shows the results of the TNF-α cytotoxicity assay for SQD-perylene and SQD-pyrene at different concentrations. Over the concentration range in Fig. 7, the cellular viability decreased by up to 40% of the control for SQD-perylene and SQD-pyrene. Hence, the nanoparticles SQD-perylene and SQD-pyrene were found to have low cytotoxicity, indicating the possibility of using SQDs for biolabeling applications.

## Conclusion

In summary, we have synthesized three families of SQDs covalently functionalized with phenanthrene, pyrene, and perylene chromophores. The PL of SQD core was red-shifted by 69 and 65 nm, and blue-shifted by 50 nm when perylene, phenanthrene, and pyrene were used as capping agents, respectively, compared to the counterpart model compound, SQD-heptene. The quantum efficiency was improved from 8% in SQD-heptene to 18% and 11% in SQD-perylene and SQD-pyrene, respectively. Therefore, the functionalization of the SQD core with aromatic fluorophores is an efficient strategy to tune the optical properties of SQDs and improve their quantum efficiency. The nanoparticles SQD-perylene and SQD-pyrene showed promising results when used for fluorescent cellular imaging with low cytotoxicity, which enables the SQDs to be used as fluorescence probes in bioimaging. We are now aiming to apply this strategy to prepare SQDs that emit in the near-IR region and use them as photodynamic therapy agents for the apoptosis of cancer cells.

## Methods

### Materials

3-Ethynyl perylene, tetraoctylammonium bromide (98%, TOAB), silicon tetrachloride (99.998%, SiCl_4_), hexachloroplatinic acid hexahydrate (Pt), lithium aluminum hydride solution (LAH, 1 M in THF), 1-ethynyl pyrene (pyrene), 3-ethynyl perylene (perylene), 9-ethynyl phenanthrene (97%, phenanthrene), and 1-heptyne (98%, heptyne) were used without additional purification. All solvents were dried by passing through MB SPS-800 (MBraun) solvent purification system with water content below 15 ppm.

### Characterization

UV-Vis absorption spectra were measured using a Shimadzu UV-1800 double beam spectrophotometer. Steady-state emission and excitation spectra and time-resolved spectra were recorded at 298 K using an Edinburgh Instruments F980. The XPS analyses were carried out with a Kratos Axis Nova spectrometer using a monochromatic Al K(alpha) source (15 mA, 14 kV). The TEM/HRTEM images were recorded using Libra 200 MC operated at 200 kV. The FTIR spectra were obtained from a Nicolet 6700 FTIR spectrometer equipped with a smart iTR diamond horizontal attenuated total reflectance (ATR).

### Photophysical measurements

All samples were prepared in HPLC grade dichloromethane with varying concentrations in the order of μM. Absorption spectra were recorded at room temperature using a Shimadzu UV-1800 double beam spectrophotometer. Molar absorptivity determination was verified by linear least-squares fit of values obtained from at least four independent solutions at varying concentrations with absorbance ranging from 4.00 × 10^−6^ to 2.00 × 10^−5^ M.

The sample solutions for the emission spectra were prepared in HPLC-grade DCM and degassed *via* bubbling nitrogen for five minutes using a quartz cuvette designed in-house. Steady-state emission and excitation spectra and time-resolved emission spectra were recorded at 298 K using an Edinburgh Instruments F980. All samples for steady-state measurements were excited at 440 nm, 360 nm, 330 nm and 310 nm while samples for time-resolved measurements were excited at 378 nm using a PDL 800-D pulsed diode laser. Emission quantum yields were determined using the optically dilute method^40^. A stock solution with absorbance of *ca*. 0.5 was prepared and then four dilutions were prepared with dilution factors between 2 and 20 to obtain solutions with absorbances of *ca*. 0.095 0.065, 0.05 and 0.018, respectively. The Beer-Lambert law was found to be linear at the concentrations of these solutions. The emission spectra were then measured after the solutions were degassed *via* bubbling nitrogen for five minutes prior to spectrum acquisition. For each sample, linearity between absorption and emission intensity was verified through linear regression analysis and additional measurements were acquired until the Pearson regression factor (R^2^) for the linear fit of the data set surpassed 0.9. Individual relative quantum yield values were calculated for each solution and the values reported represent the slope value. The equation Φ_s_ = Φ_r_(*A*_*r*_*/A*_*s*_)(*I*_*s*_*/I*_*r*_)(*n*_s_/*n*_r_)^41^ was used to calculate the relative quantum yield of each of the sample, where Φ_r_ is the absolute quantum yield of the reference, *n* is the refractive index of the solvent, *A* is the absorbance at the excitation wavelength, and *I* is the integrated area under the corrected emission curve. The subscripts s and r refer to the sample and reference, respectively. A solution of quinine sulfate in 0.5 M H_2_SO_4_ (Φ_r_ = 54.6%) was used as external ref.^42^.

### Synthesis and purification of functionalized SQDs

The synthesis of SQDs functionalized with conjugated aromatic linkages was carried out using Tilley’s method^12,43^. All experiments were performed under argon atmosphere using a glovebox. In a typical experiment, 1.5 g of TOAB and 150 µL of SiCl_4_ were dissolved in 100 mL of dry toluene by stirring for 45 minutes. An excess amount of lithium aluminum hydride (LAH) solution was then added, and the mixture was further stirred for 3 hours to produce H-terminated SQDs. Anhydrous methanol was then added to quench the excess of LAH until no further effervescence was observed. The passivation of H-terminated SQDs was carried out by reacting the SQDs with 4 mL of heptyne to produce SQD-heptene using 100 µL of 0.1 M Pt catalyst in methanol. The latter procedure was repeated using phenanthrene (500 mg), pyrene (500 mg), and perylene (500 mg) as capping agents to produce SQD-phenanthrene, SQD-pyrene, and SQD-perylene, respectively (Fig. 1). The resulting capped SQDs were purified by dialysis against water to removed inorganic salts, and then DCM to remove organic impurities (MWCO of 1 KDa, Spectra/Por® 6 Standard RC Pre-wetted Dialysis Tubing, diameter 29 mm).

### Fluorescent Imaging

The HeLa cells were cultured one day prior to imaging at a volume of 1 × 10^5^ cells/well on a 6-well culture plate with the medium (Dulbecco’s modified Eagle’s medium (DMEM), 10% fetal calf serum and 4 mM L-glutamine) at 37 °C and 5% CO_2_. On the day of imaging, cells were grown to 80% confluence and they were incubated with 50–100 µg/ml of functionalized SQDs dispersed in phosphate-buffered saline (PBS) for 3 hrs. Immediately before imaging, the medium was removed and HeLa cells were washed 3 × using fresh PBS solution. The cells were then imaged using a confocal microscope (Zeiss LSM 510 Duo Confocal).

### Cytotoxicity (TNF-α)

TNF-α assay was performed to evaluate the cytotoxicity of SQD-pyrene and SQD-perylene. Cells were cultured in 96-well microplates in a humidified atmosphere (37 °C and 5% CO_2_) to 70 − 80% confluence. The cells were seeded at a concentration of 5 × 10^4^ cells/well in 100 µl of culture medium (1 µg/ml of actinomycin C1 and various amounts of TNF-α) and different concentrations of SQDs. The cells were incubated for 24 hours at these conditions. Afterwards, 10 µl of Cell Proliferation Reagent WST-1 was added and incubated for another 4 hours, and they were finally shaken for 1 minute. The absorbance of the sample was measured against the background control as blank using a microplate (ELISA) reader at wavelength of 450 nm.

### Data availability

All data generated or analysed during this study are included in this published article (and its Supplementary Information files).

## Electronic supplementary material

Supplementary Information
