# Supplementary material for: Tuning the Optical Properties of Silicon Quantum Dots via Surface Functionalization with Conjugated Aromatic Fluorophores
Source: Sci Rep. 2018 Feb 14;8:3050. doi: 10.1038/s41598-018-21181-8 (PMC5813013; doi:10.1038/s41598-018-21181-8)
Supplement: Supplementary file 1 — Supplementary Information [file 41598_2018_21181_MOESM1_ESM.pdf]

Tuning the Optical Properties of Silicon Quantum Dots via Surface Functionalization with Conjugated Aromatic Fluorophores

Mohammed Abdelhameed,[a] Diego Rota Martira,[b] Shalimar Chen,[c] William Z. Xu,[a] Olabode O. Oyeneye,[a] Subrata Chakrabarti,[c] Eli Zysman-Colman [b] and Paul A. Charpentier [a]

Figure S1. FTIR spectra of 3-Ethynylperylene, 9-Ethynylphenanthrene, and 1-Ethynylpyrene. **3**
Figure S2. XPS spectra of C 1S for SQD-pyrene. **4**
Figure S3. UV-Vis absorption, excitation, and emission spectra of SQD-heptene collected in degassed DCM at 298 K (λ_{ex} = 360 nm). **5**
Figure S4. UV-Vis absorption, excitation, and emission spectra of SQD-heptene collected in degassed DCM at 298 K (λ_{ex} = 315 nm). **6**
Figure S5. UV-Vis spectra, excitation, and emission spectra of Perylene and SQD-perylene collected in degassed DCM at 298 K (λ_{ex} = 360 nm). **7**
Figure S6. UV-Vis spectra, Excitation, and Emission spectra of Pyrene and SQD-pyrene collected in degassed DCM at 298 K (λ_{ex} = 360 nm). **8**
Figure S7. UV-Vis spectra, excitation, and emission spectra of Phenanthrene and SQD-phenanthrene collected in degassed DCM at 298 K (λ_{ex} = 315 nm). **9**
Figure S8. Emission spectra of SQD-perylene collected in degassed DCM at 298 K upon photoexcitation at 360 nm and at 450 nm. **10**
Figure S9. Emission spectra of SQD-pyrene collected in degassed DCM at 298 K upon photoexcitation at 360 nm and at 348 nm. **11**
Figure S10. Emission spectra of SQD-phenanthrene collected in degassed DCM at 298 K upon photoexcitation at 360 nm and at 315 nm. **12**
Figure S11. Emission spectra of SQD-pyrene collected in degassed DCM at 298 K at a concentration of 100 μM, 50 μM, 10 μM and 1 μM (λ_{ex} = 360 nm). **13**
Figure S12. Emission spectra of Pyrene collected in degassed DCM at 298 K at a concentration of 1.5 mM, 550 μM, 368 μM and 176 μM (λ_{ex} = 360 nm). **14**
Figure S13. Emission decay of SQD-heptene monitored at 448 nm collected in degassed DCM at 298 K (λ_{ex} = 378 nm). **14**
Figure S14. Emission decay of perylene monitored at 467 nm collected in degassed DCM at 298 K (λ_{ex} = 378 nm). **15**
Figure S15. Emission decay of SQD-perylene monitored at 519 nm collected in degassed DCM at 298 K (λ_{ex} = 378 nm). **15**
Figure S16. Emission decay of pyrene monitored at 384 nm collected in degassed DCM at 298 K (λ_{ex} = 378 nm). **16**
Figure S17. Emission decay of SQD-pyrene monitored at 394 nm collected in degassed DCM at 298 K (λ_{ex} = 378 nm). **16**

Figure S18. Emission decay of phenanthrene monitored at 376 nm collected in degassed DCM at 298 K (λ_{ex} = 378 nm). ……………………………………………………………….. **17**
Figure S19. Emission decay of SQD-phenanthrene monitored at 446 nm collected in degassed DCM at 298 K (λ_{ex} = 378 nm). ……………………………………………….. **17**

Figure S20. Emission spectra of Perylene, Pyrene, Phenanthrene, SQD-perylene, SQD-pyrene, and SQD-phenanthrene. …………………………………………………………. **18**

Figure S21. Emission spectra of SQD-perylene (blue) and SQD-pyrene (orange) in water…….………………………………………………………………………… **19**

Figure S22. Confocal images of HeLa cells for DIC images (panels A and D), fluorescence images (panels B and E), and merged images (panels C and F)…………… **20**

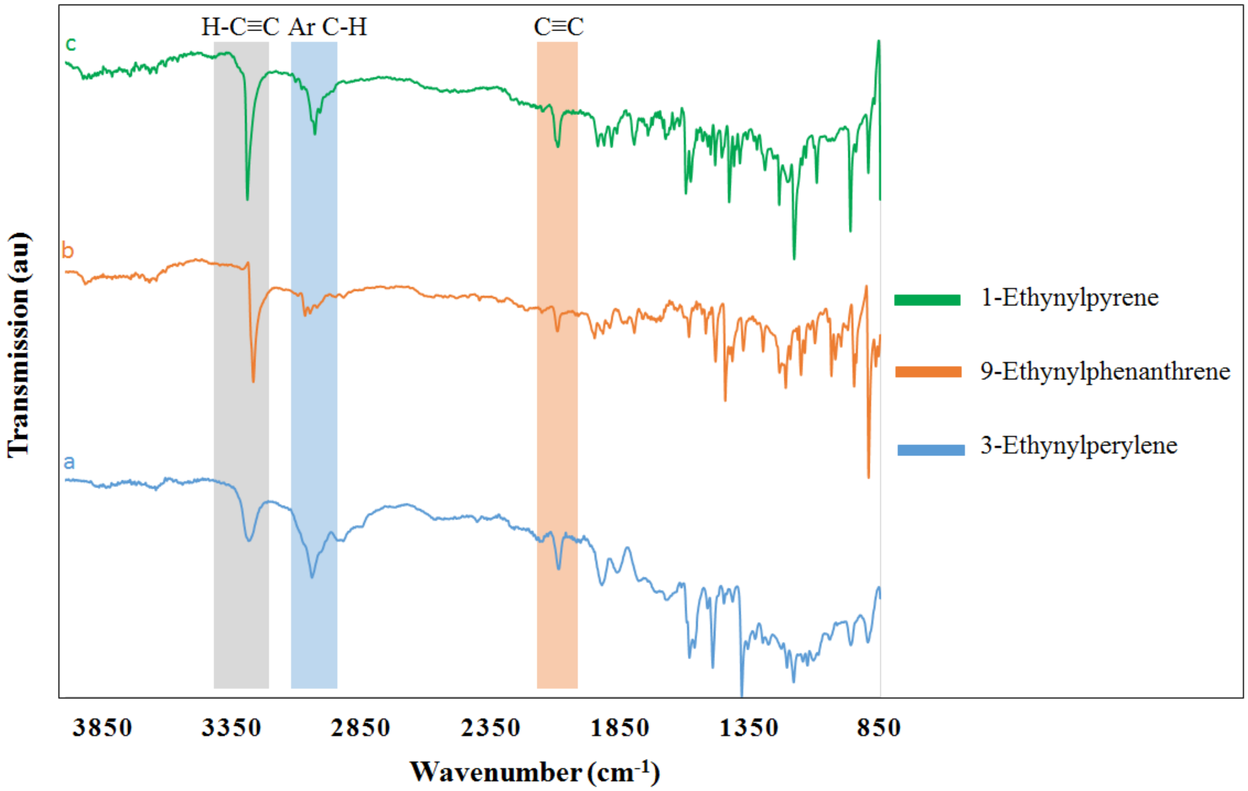

Figure S1. FTIR spectra of 3-Ethynylperylene (a), 9-Ethynylphenanthrene (b), and 1-Ethynylpyrene (c).

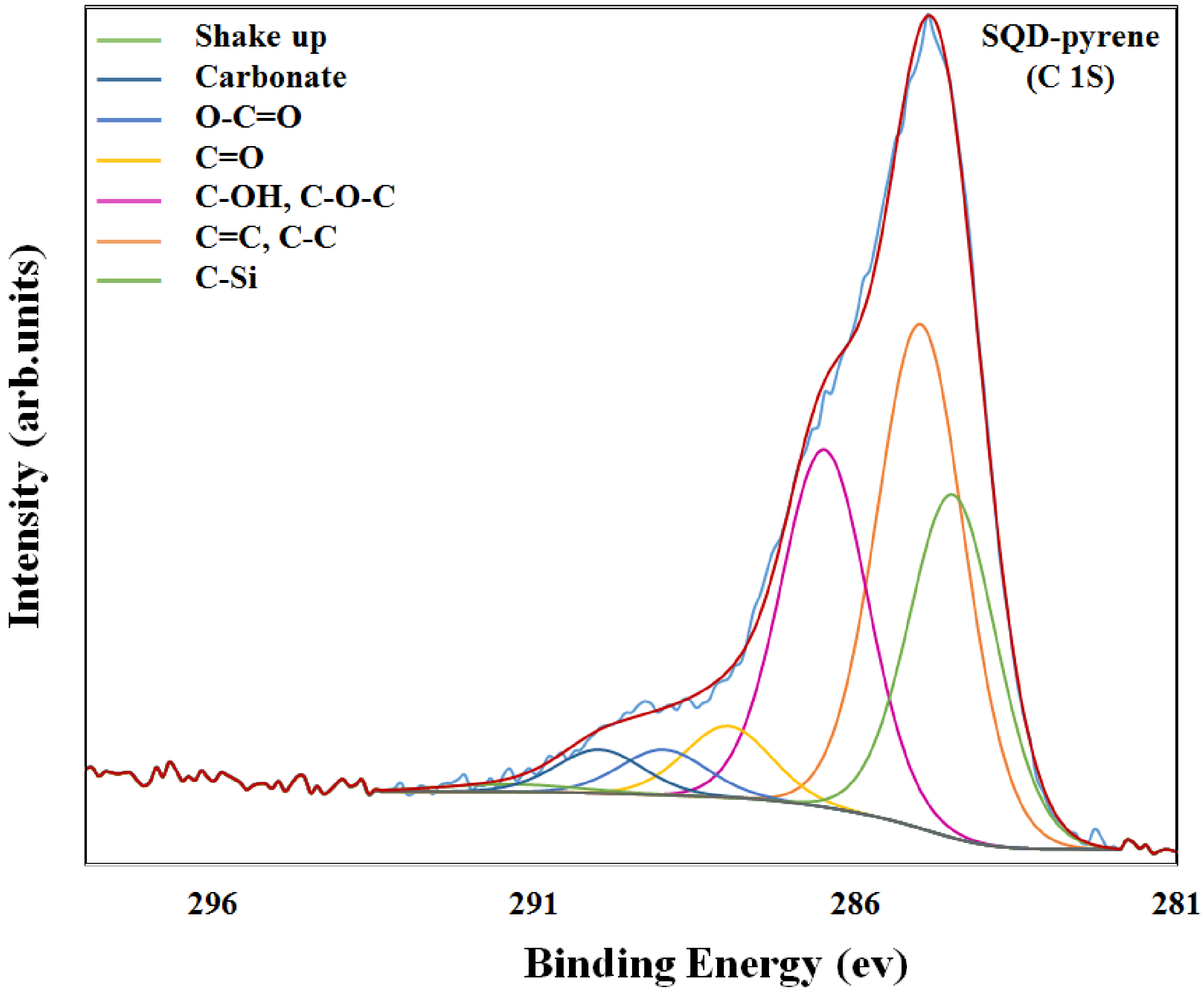

Figure S2. XPS spectra of C 1S for SQD-pyrene

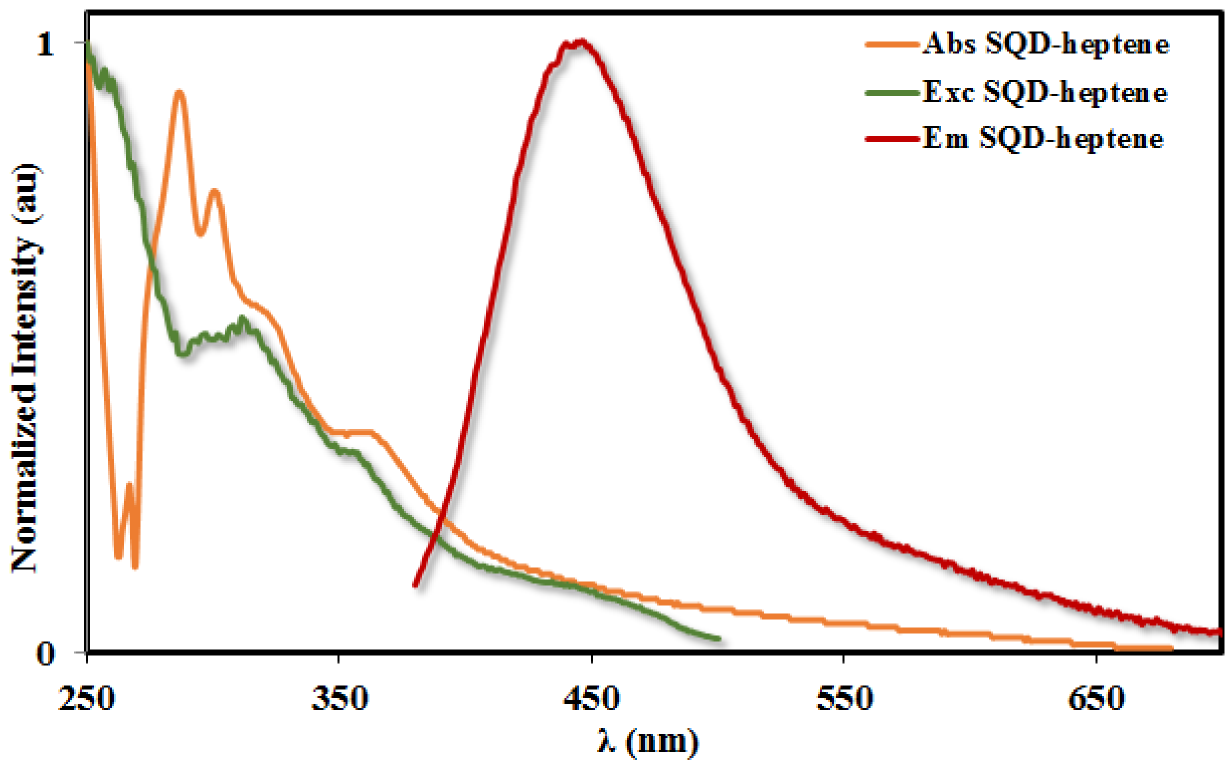

Figure S3. UV-Vis absorption, excitation (λ_{em} = 448 nm), and emission spectra of SQD-heptene collected in degassed DCM at 298 K (λ_{ex} = 360 nm).

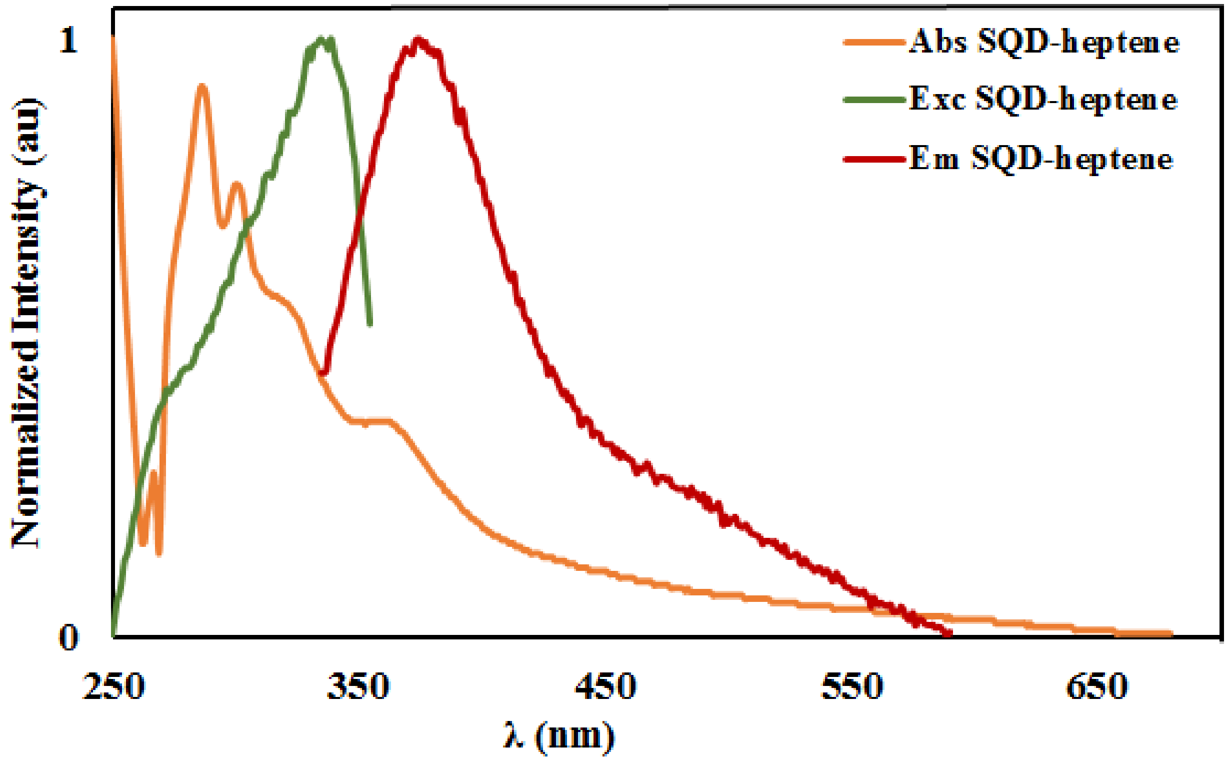

Figure S4. UV-Vis absorption, excitation (λ_{em} = 374 nm), and emission spectra of SQD-heptene collected in degassed DCM at 298 K (λ_{ex} = 315 nm).

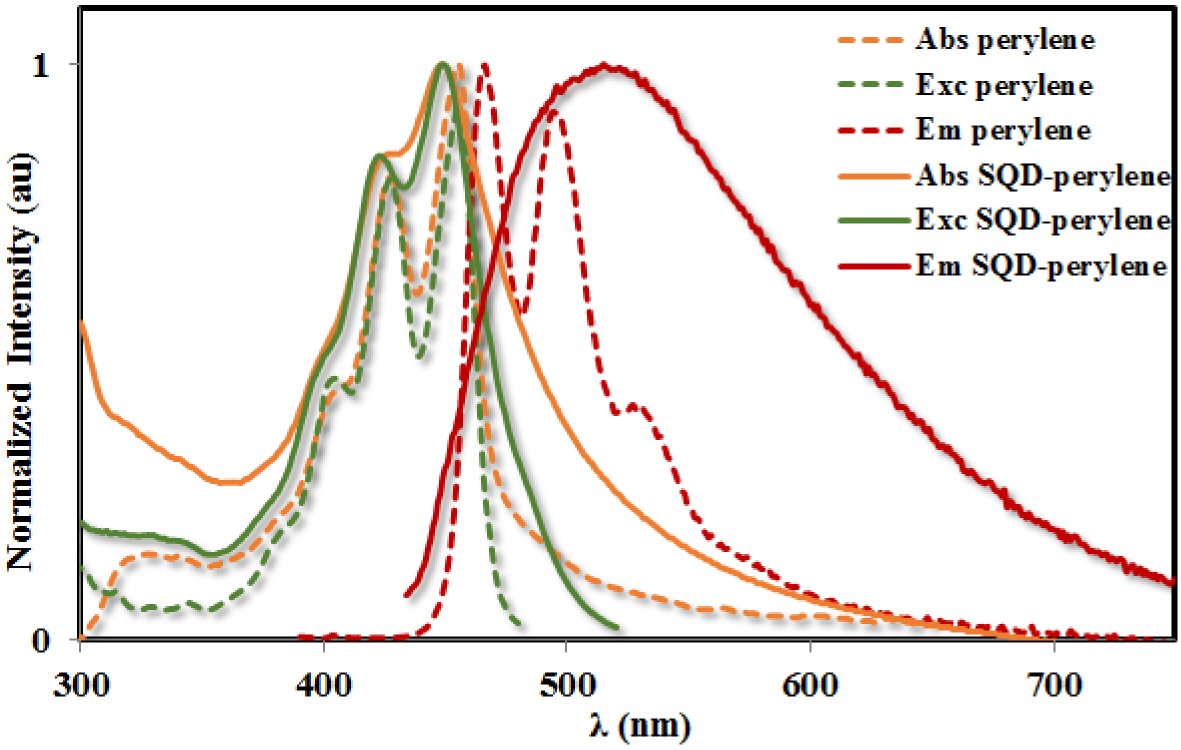

Figure S5. UV-Vis spectra of perylene (dotted orange line) and SQD-perylene (solid orange line); excitation spectra of perylene (dotted green line, λ_{em} = 467 nm) and SQD-perylene (solid green line, λ_{em} = 518 nm); and emission spectra of perylene (dotted red line) and SQD-perylene (solid red line) collected in degassed DCM at 298 K (λ_{ex} = 360 nm).

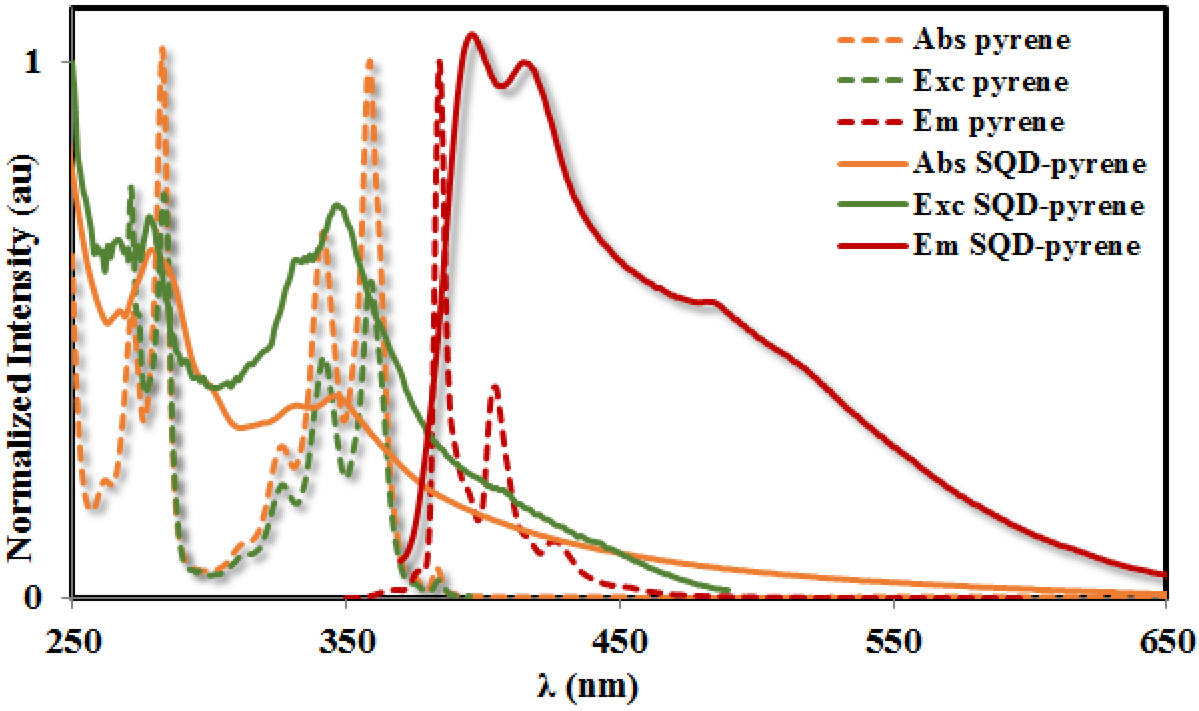

Figure S6. UV-Vis spectra of pyrene (dotted orange line) and SQD-pyrene (solid orange line); excitation spectra of pyrene (dotted green line, λ_{em} = 384 nm) and SQD-pyrene (solid green line, λ_{em} = 397 nm); and emission spectra of pyrene (dotted red line) and SQD-pyrene (solid red line) collected in degassed DCM at 298 K (λ_{ex} = 360 nm).

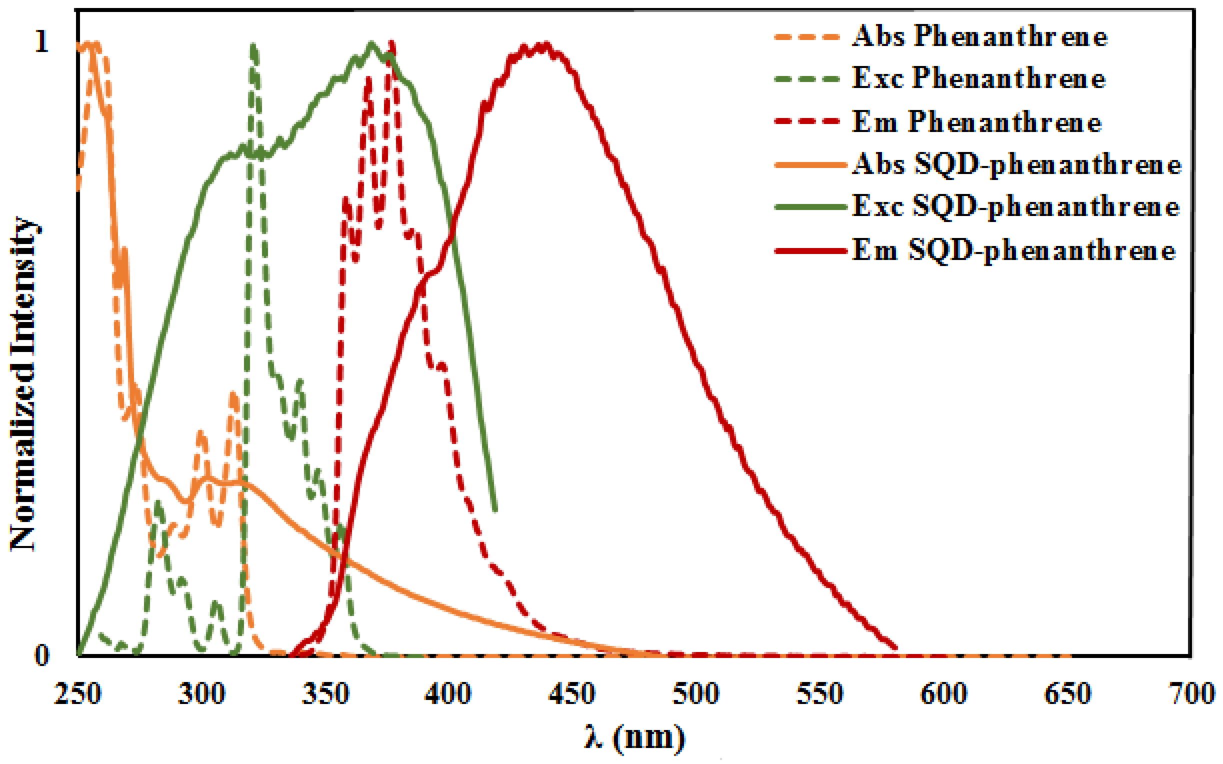

Figure S7. UV-Vis spectra of phenanthrene (dotted orange line) and SQD-phenanthrene (solid orange line); excitation spectra of Phenanthrene (dotted green line, λ_{em} = 377 nm) and SQD-phenanthrene (solid green line, λ_{em} = 447 nm); and emission spectra of phenanthrene (dotted red line) and SQD-phenanthrene (solid red line) collected in degassed DCM at 298 K (λ_{ex} = 315 nm).

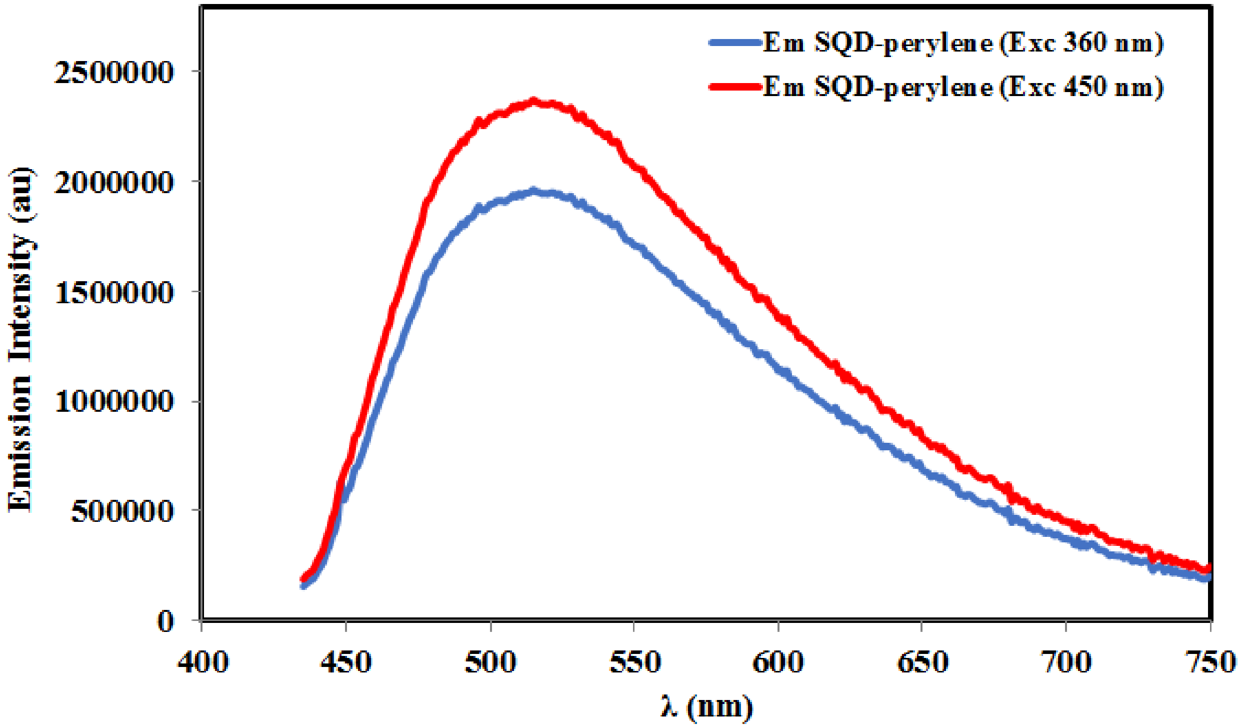

Figure S8. Emission spectra of SQD-perylene collected in degassed DCM at 298 K upon photoexcitation at 360 nm (light-blue line) and at 450 nm (red line).

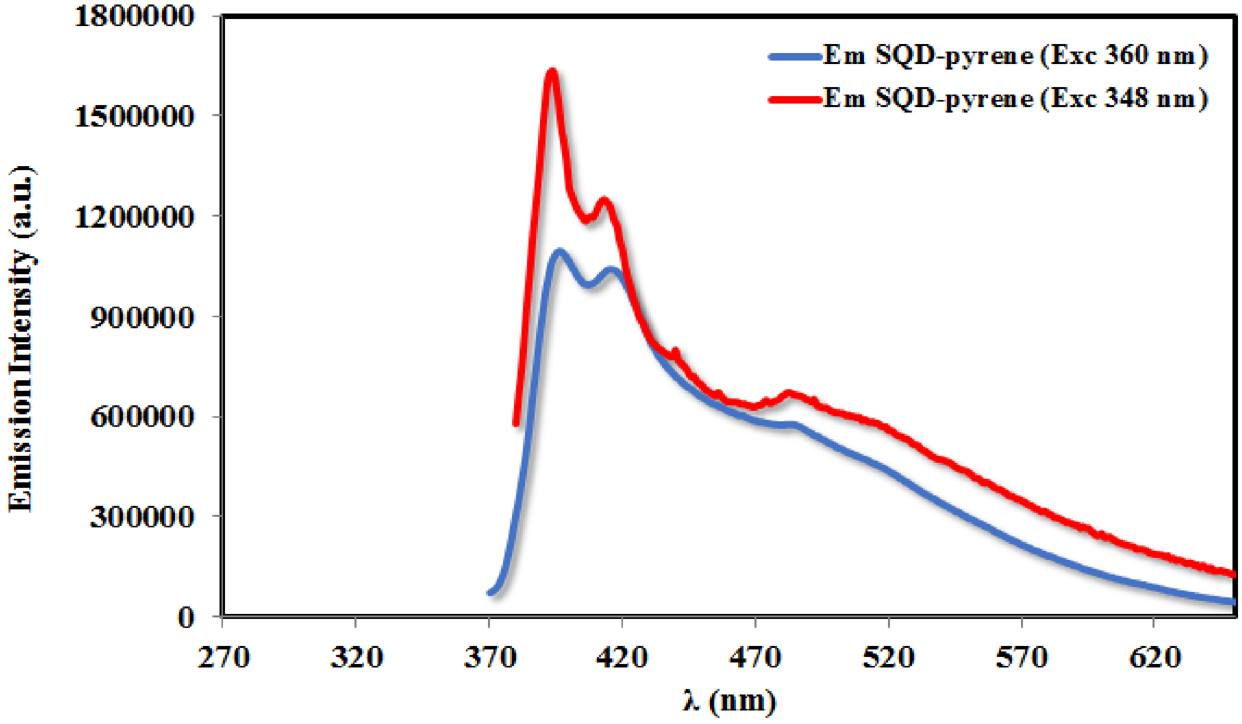

Figure S9. Emission spectra of SQD-pyrene collected in degassed DCM at 298 K upon photoexcitation at 360 nm (light-blue line) and at 348 nm (red line).

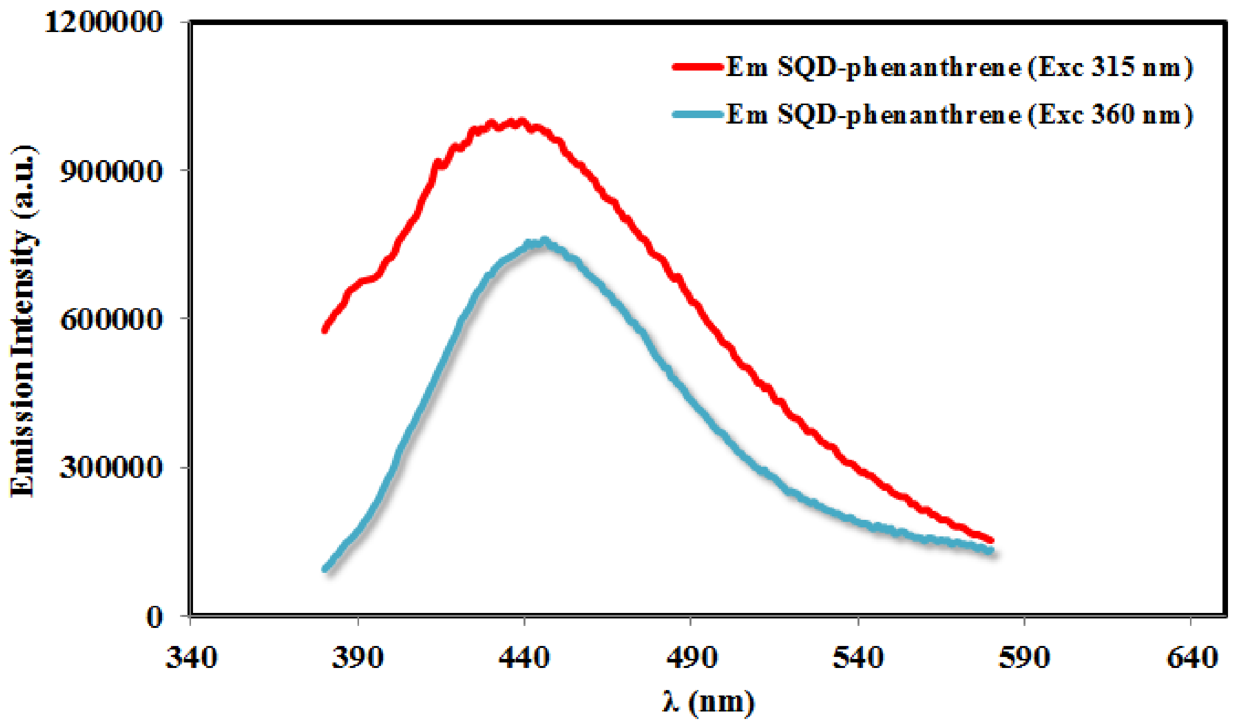

Figure S10. Emission spectra of SQD-phenanthrene collected in degassed DCM at 298 K upon photoexcitation at 360 nm (light-blue line) and at 315 nm (red line).

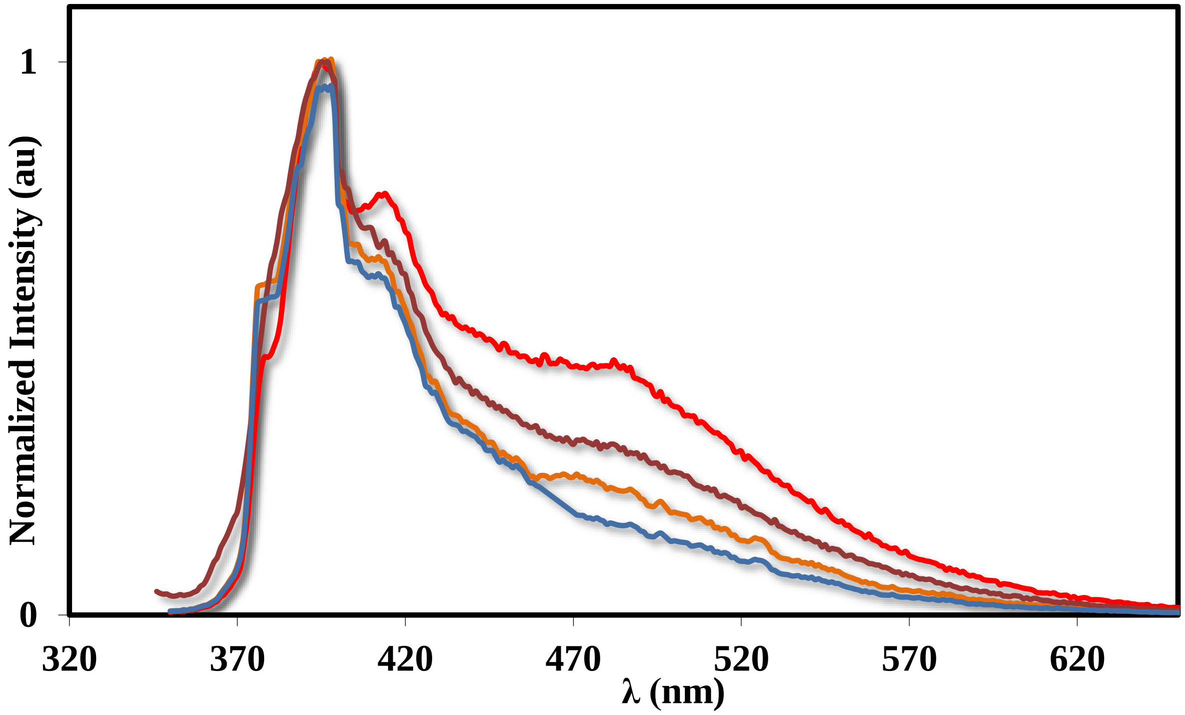

Figure S11. Emission spectra of SQD-pyrene collected in degassed DCM at 298 K at a concentration of 100 μM (red line), 50 μM (purple line), 10 μM (orange line) and 1 μM (blue line) (λ_{ex} = 360 nm).

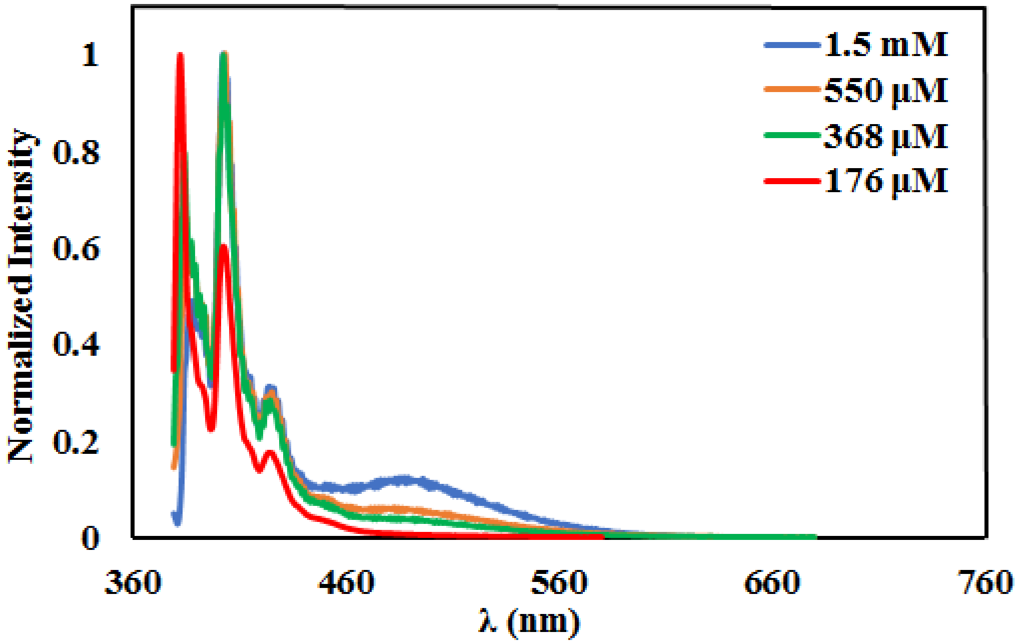

Figure S12. Emission spectra of pyrene collected in degassed DCM at 298 K at a concentration of 1.5 mM (blue line), 550 µM (orange line), 368 µM (green line) and 176 µM (red line) (λ_{ex} = 360 nm).

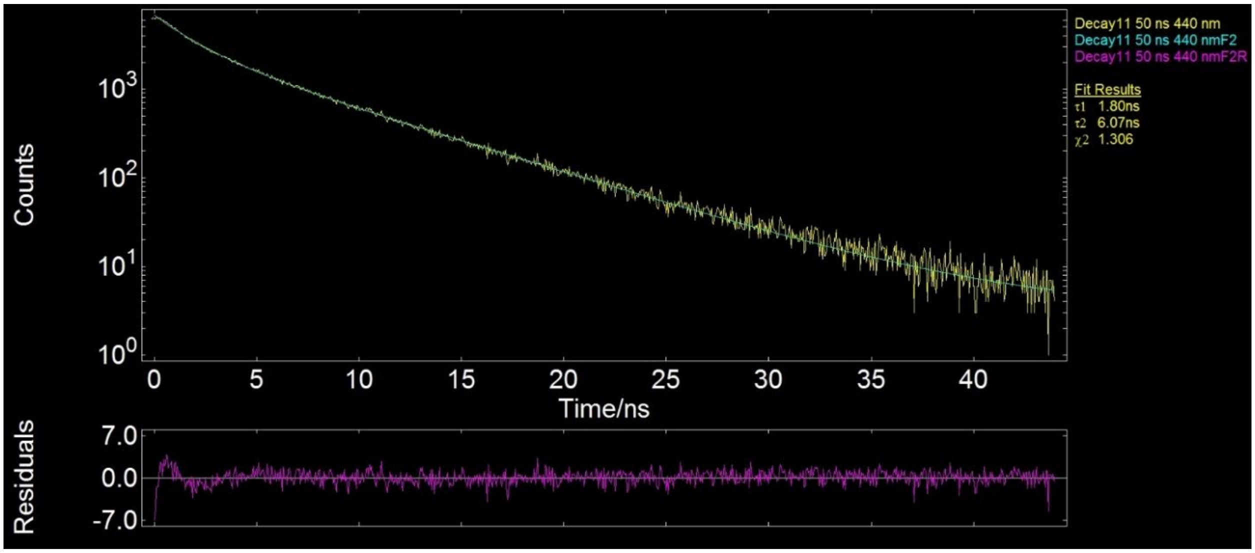

Figure S13. Emission decay of SQD-heptene monitored at 448 nm collected in degassed DCM at 298 K (λ_{ex} = 378 nm).

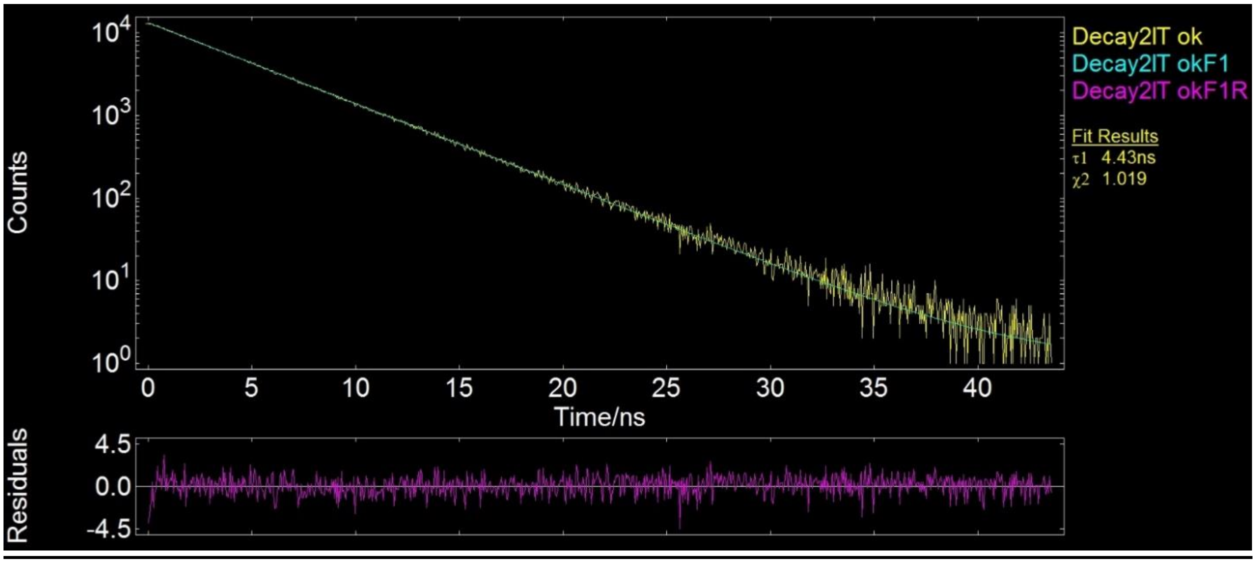

Figure S14. Emission decay of perylene monitored at 467 nm collected in degassed DCM at 298 K (λ_{ex} = 378 nm).

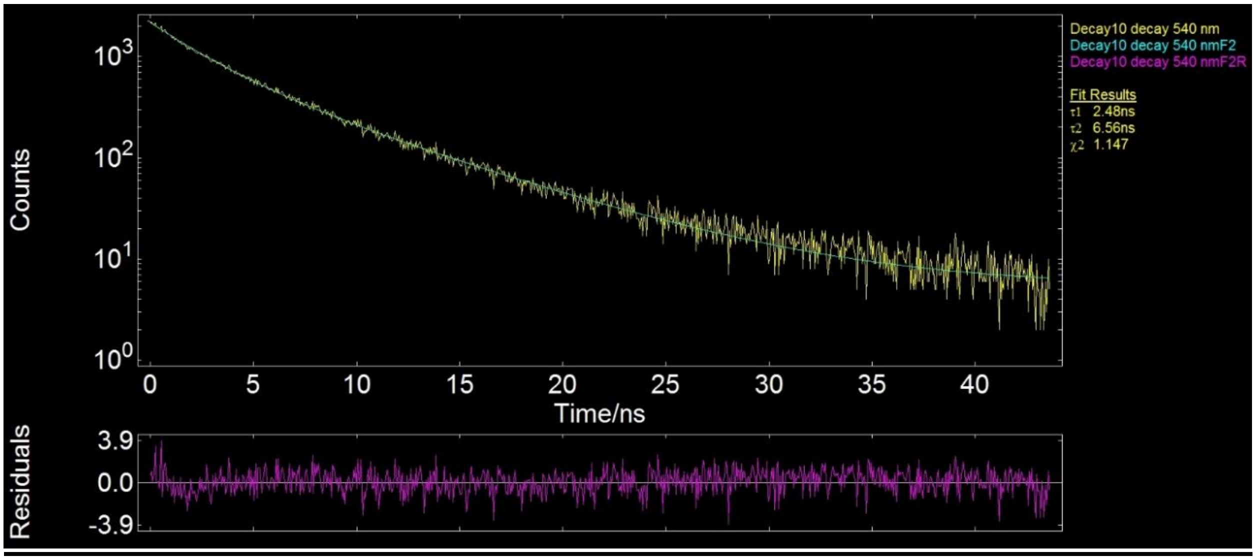

Figure S15. Emission decay of SQD-perylene monitored at 519 nm collected in degassed DCM at 298 K (λ_{ex} = 378 nm).

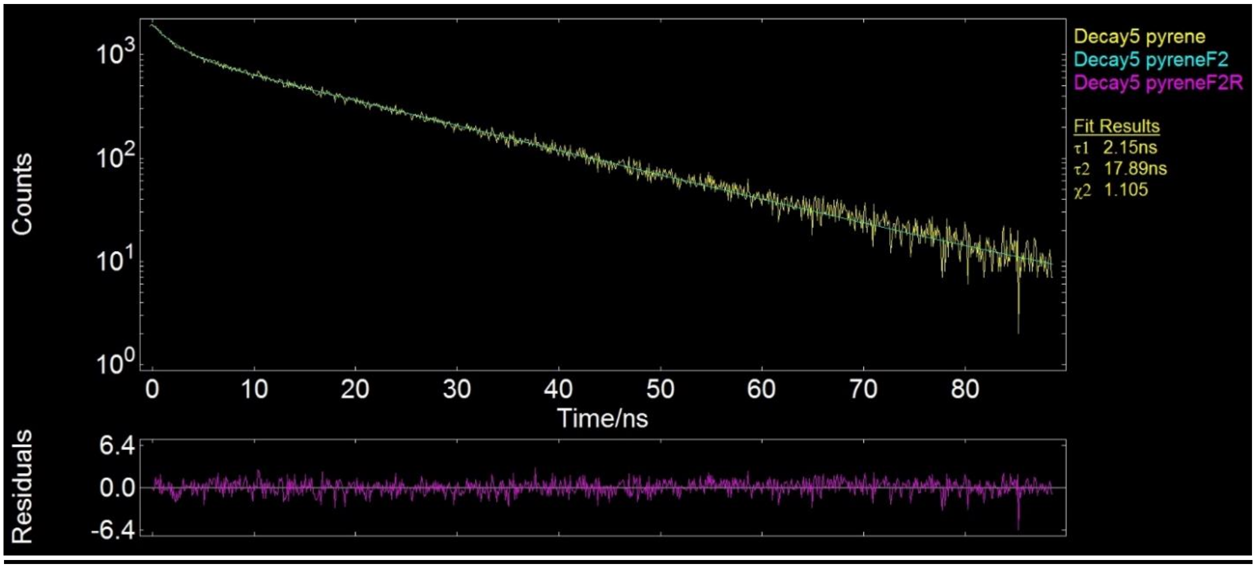

Figure S16. Emission decay of pyrene monitored at 384 nm collected in degassed DCM at 298 K (λ_{ex} = 378 nm).

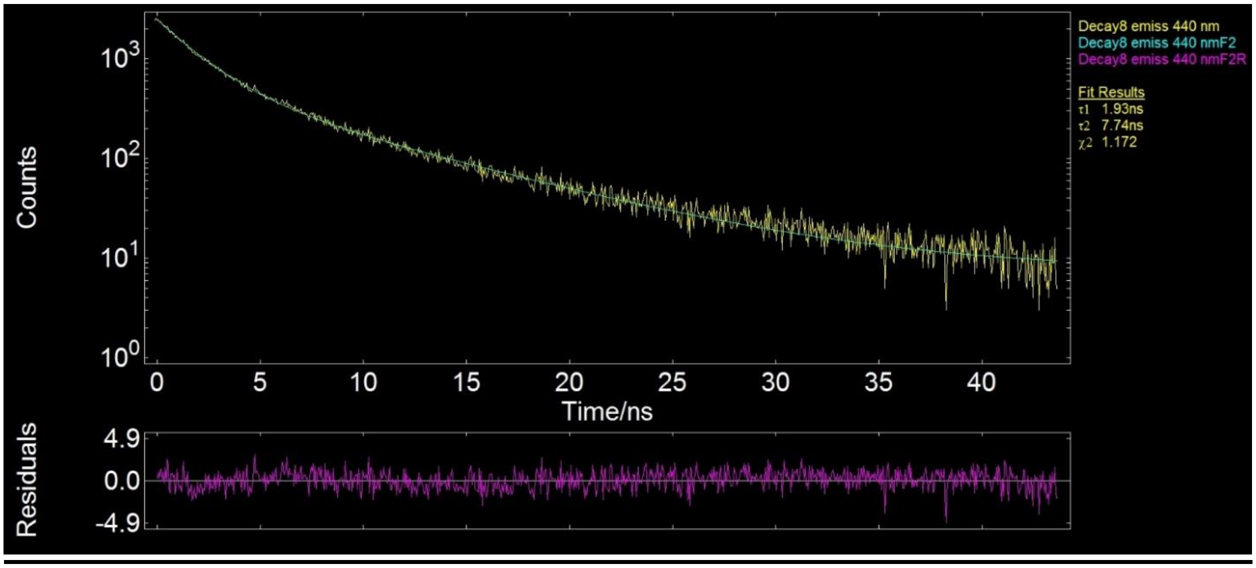

Figure S17. Emission decay of SQD-pyrene monitored at 394 nm collected in degassed DCM at 298 K (λ_{ex} = 378 nm).

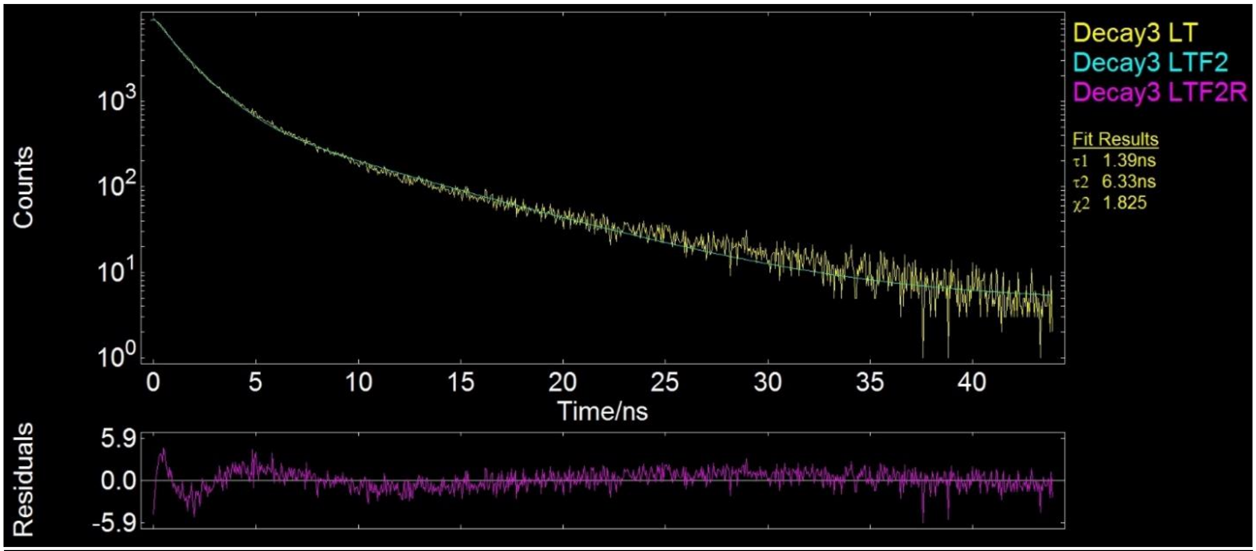

Figure S18. Emission decay of phenanthrene monitored at 376 nm collected in degassed DCM at 298 K (λ_{ex} = 378 nm).

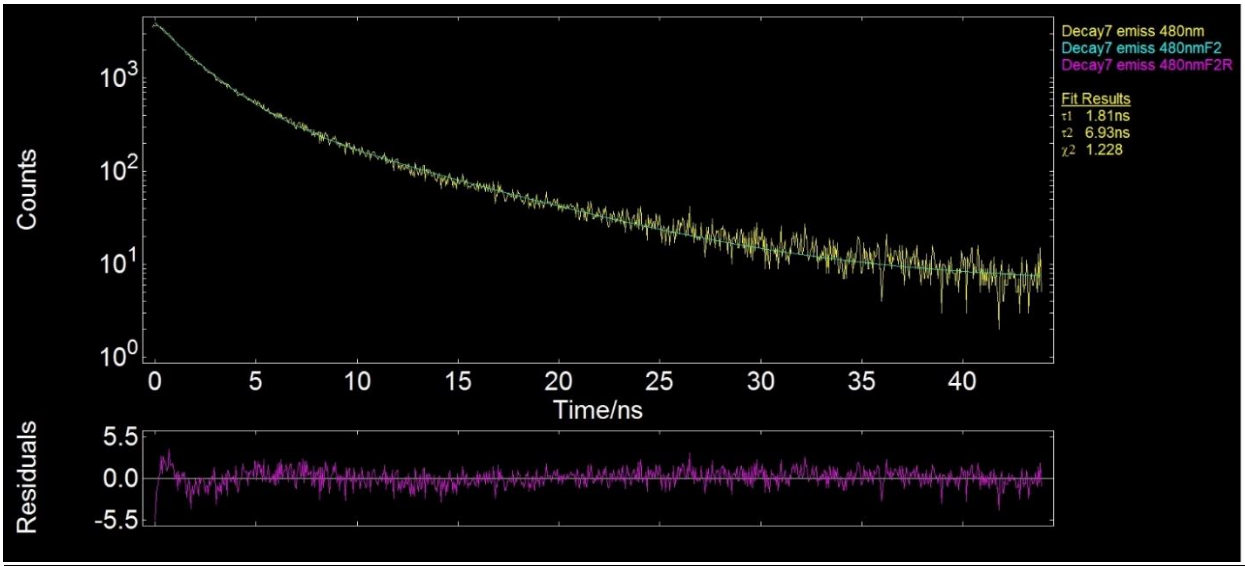

Figure S19. Emission decay of SQD-phenanthrene monitored at 446 nm collected in degassed DCM at 298 K (λ_{ex} = 378 nm).

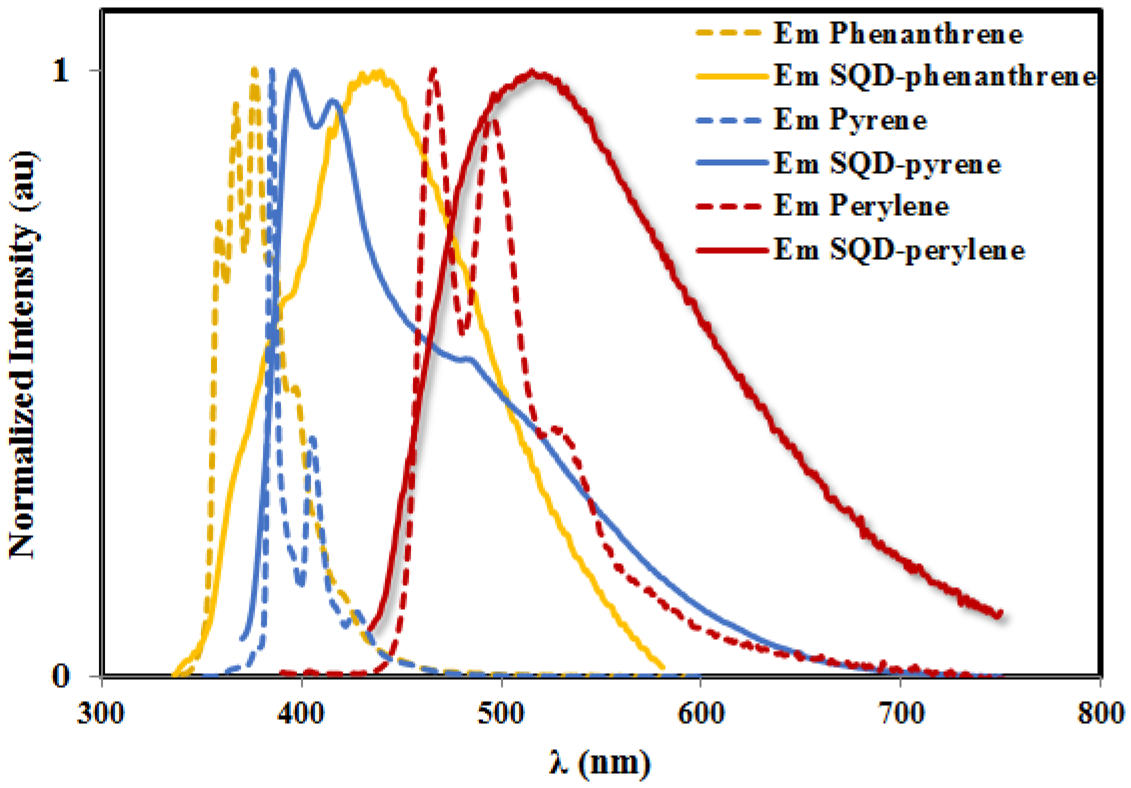

Figure S20. Emission spectra of perylene (λ_{ex} = 360 nm, dotted red line) and SQD-perylene (λ_{ex} = 360 nm, solid red line); pyrene (λ_{ex} = 360 nm, dotted blue line) and SQD-pyrene (λ_{ex} = 360 nm, solid blue-line); phenanthrene (λ_{ex} = 315 nm, dotted yellow line) and SQD-phenanthrene (λ_{ex} = 315 nm, solid yellow line) collected in degassed DCM at 298 K.

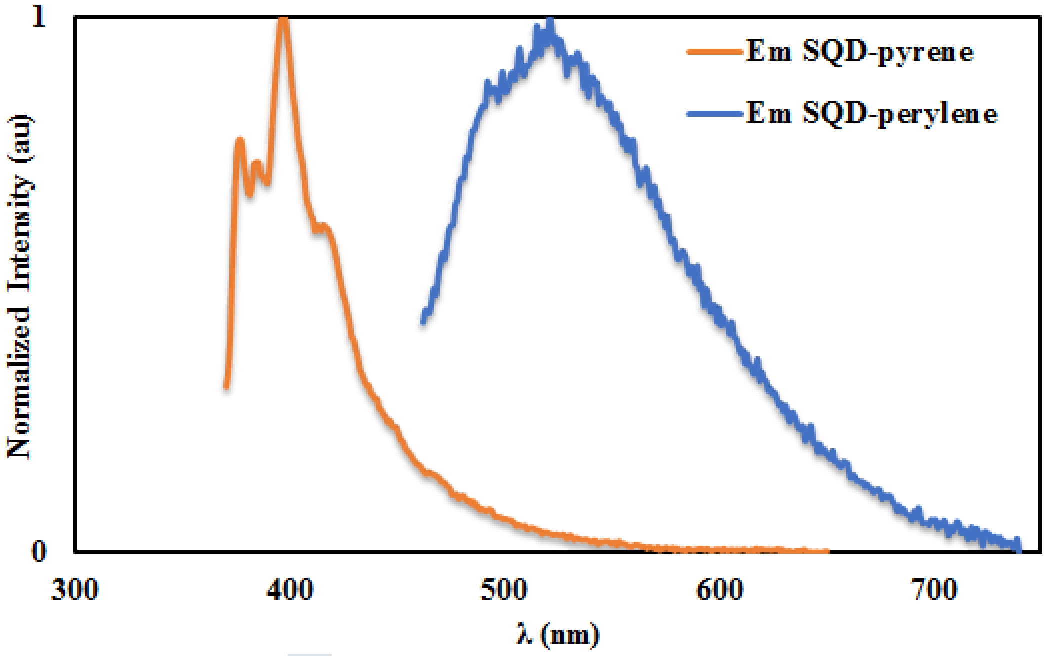

Figure S21. Emission spectra of SQD-perylene (λ_{ex} = 440 nm, blue) and SQD-pyrene (λ_{ex} = 350 nm, orange) in water.

Fluorescent cellular imaging study

In figure 5, we utilized a confocal microscope to collect the photoluminescence obtained from HeLa cells that were incubated with SQD-perylene and SQD-pyrene. Cells imaging was collected at two different channels of 400-450 and 500-550 nm in the presence of a combination of short- and long-pass filters, and at an excitation wavelength of 405 nm. When the emission was monitored using the 440-450 nm channel, a blue emission was obtained as shown in figure 5 while other emission wavelengths were filtered out. On the other hand, figure S22 below shows the green emission collected when the 500-550 nm channel was utilized. The broad emission spectrum of both SQD-perylene and SQD-pyrene has enabled to collect blue and green emission color when the channels 400-450 and 500-550 nm were utilized, respectively.

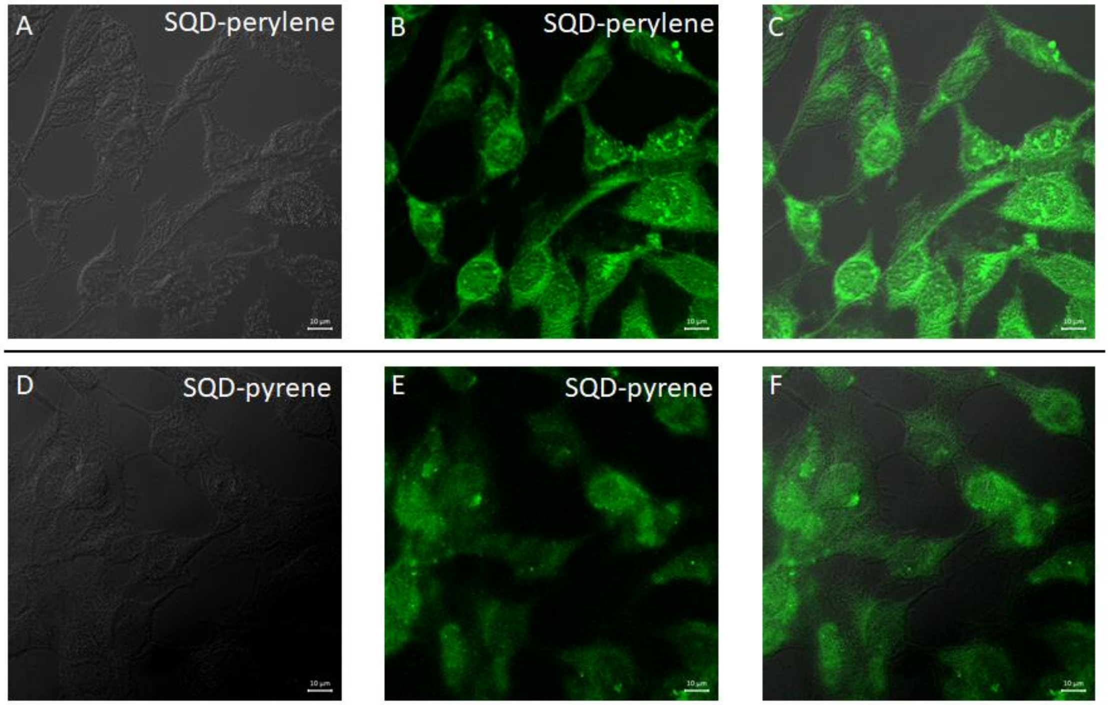

Figure S22. Confocal images of HeLa cells for DIC images (panels A and D), fluorescence images (panels B and E), and merged images (panels C and F).
